# Arginine-, d-arginine-vasopressin, and their *inverso* analogues in micellar and liposomic models of cell membrane: CD, NMR, and molecular dynamics studies

**DOI:** 10.1007/s00249-015-1071-4

**Published:** 2015-08-20

**Authors:** Emilia A. Lubecka, Emilia Sikorska, Dariusz Sobolewski, Adam Prahl, Jiřina Slaninová, Jerzy Ciarkowski

**Affiliations:** Faculty of Chemistry, University of Gdańsk, Wita Stwosza 63, 80-308 Gdańsk, Poland; Institute of Organic Chemistry and Biochemistry, Academy of Sciences of the Czech Republic, 166 10 Prague, Czech Republic

**Keywords:** Antidiuretic agonists, Anionic–zwitterionic micelles, Liposomes, *Inverso* analogues

## Abstract

We describe the synthesis, pharmacological properties, and structures of antidiuretic agonists, arginine vasopressin (AVP) and [d-Arg^8^]-vasopressin (DAVP), and their *inverso* analogues. The structures of the peptides are studied based on micellar and liposomic models of cell membranes using CD spectroscopy. Additionally, three-dimensional structures in mixed anionic–zwitterionic micelles are obtained using NMR spectroscopy and molecular dynamics simulations. NMR data have shown that AVP and DAVP tend to adopt typical of vasopressin-like peptides *β*-turns: in the 2–5 and 3–6 fragments. The *inverso*-analogues also adopt *β*-turns in the 3–6 fragments. For this reason, their inactivity seems to be due to the difference in side chains orientations of Tyr^2^, Phe^3^, and Arg^8^, important for interactions with the receptors. Again, the potent antidiuretic activity of DAVP can be explained by CD data suggesting differences in mutual arrangement of the aromatic side chains of Tyr^2^ and Phe^3^ in this peptide in liposomes rather than of native AVP. In the presence of liposomes, the smallest conformational changes of the peptides are noticed with DPPC and the largest with DPPG liposomes. This suggests that electrostatic interactions are crucial for the peptide–membrane interactions. We obtained similar, probably active, conformations of the antidiuretic agonists in the mixed DPC/SDS micelles (5:1) and in the mixed DPPC/DPPG (7:3) liposomes. Thus it can be speculated that the anionic–zwitterionic liposomes as well as the anionic–zwitterionic micelles, mimicking the eukaryotic cell membrane environment, partially restrict conformational freedom of the peptides and probably induce conformations resembling those of biologically relevant ones.

**Electronic supplementary material:**

The online version of this article (doi:10.1007/s00249-015-1071-4) contains supplementary material, which is available to authorized users.

## Introduction

The neurohypophyseal hormone (NPH) arginine vasopressin, CYFQNCPRG-NH_2_ (AVP), is a circulating endogenous nonapeptide with a disulphide bridge between Cys^1^ and Cys^6^. It exhibits a renal antidiuretic effect by supporting conservation of osmolality and the volume of body fluids, controlling urine volume and renal sodium excretion (Barlow 2002; Warne et al. 2001). AVP also displays pressor and oxytocic activities (Barberis et al. 1998) and controls secretion of the adrenocorticotropic hormone (ACTH) (Jard et al. 1986). Those AVP effects are mediated by four receptors, namely the renal (V2), vascular (V1a), oxytocic (OT), and pituitary (V1b) receptors, which belong to class A of the G protein-coupled receptor (GPCR) superfamily and are membrane-spanning proteins (Palczewski et al. 2000). Moreover, recent studies have shown that vasopressin has unique effects on normal expression of species-typical social behavior, communication, and rituals, and may turn out to be an effective remedy for the treatment for autism’s repetitive and affiliative behaviors (Newschaffer et al. 2007; Insel et al. 1999).

There is evidence to supporting the membrane-bound pathway for the interaction of a ligand with its cognate GPCRs (Moroder et al. 1993; Langelaan and Rainey 2010; Langelaan et al. 2011). In this mechanism, adsorption of the ligand to the cell membrane is followed by a two-dimensional diffusion process, whereby the ligand binds to and activates the receptor (Schwyzer 1995; Mierke and Giragossian 2001). The conformation of the ligand, in its pre-associated state with the cell membrane, is thought to resemble a bioactive conformation, thus reducing the entropic penalty associated with the ligand–receptor recognition. In concordance with this mechanism, conformational and dynamic properties of ligands should be examined in a membrane-mimicking environment to get a better understanding of molecular features involved in their interactions with target receptors. As appropriate models of eukaryotic cell membrane can be considered phosphatidylcholine lipids, especially those with addition of a small amount of lipids having negatively charged head groups, to mimic electrostatic properties of the plasma membrane characterized by a slight prevalence of a negative charge. One of the popular model membranes is 1,2-dipalmitoyl-*sn*-glycero-3-phosphatidylcholine (DPPC) with addition of a small amount of 1,2-dipalmitoyl-*sn*-glycero-3-phosphatidylglycerol (DPPG) (Hirsh et al. 1996; Sikorska et al. 2012; Ergun et al. 2014). In water, the lipids undergo self-organization into closed bilayers called vesicles or liposomes. The big size of these objects and the resulting slow tumbling make them unsuitable for solution NMR (Warschawski et al. 2011). On the other hand, detergents are most useful in NMR-aided structural studies in solution because of their multiple roles in such steps as solubilization, purification, transfer, renaturation, and reconstitution of membrane proteins and peptides (Warschawski et al. 2011). The properties of peptide–detergent complexes strongly depend on the physicochemical properties of the detergents. Thus, it is important to choose detergents with small aggregation numbers to obtain small, fast-tumbling peptide–detergent complexes and intensify the peptide signal because of the reduced surfactant proportion. Therefore, in high-resolution NMR studies of peptide–membrane interactions, dodecylphosphocholine (DPC) and sodium dodecyl sulphate (SDS) (Wymore et al. 1999; Beswick et al. 1998b; Strandberg and Ulrich 2004; MacKenzie et al. 1997; Pages et al. 2009) are commonly used as a membrane-mimicking environment. The zwitterionic DPC micelle constitutes a realistic model of membrane interfaces, but its high curvature may restrict their use to relatively small peptides or proteins (Beswick et al. 1998a). A small negative charge on the surface of the micelle can be generated by addition of an anionic surfactant, SDS. In addition, SDS enhances the peptide solubility in an aqueous micelle solution (Sankararamakrishnan 2006).

It is known that the main structural features of AVP-like hormones are *β*-turns at positions 3,4 and/or 4,5 (Liwo et al. 1996). The *β*-turn in the Cys^6^–Gly^9^ C-terminal tail contributes to enhancement of the antidiuretic activity, though it is not crucial for its appearance (Sikorska et al. 2010). Moreover, the side chains of Tyr^2^ and Phe^3^ of vasopressin-like hormones are crucial for their activities (Manning et al. 1982; van Kesteren et al. 1992; Hlavacek 1987). The phenylalanine residue in position 3 of AVP is involved mainly in recognition of the hormone and its binding to receptors (Hlavacek 1987). In turn, the tyrosine residue in position 2 of AVP participates in initiating the pressor response of AVP (Hlavacek 1987). The change of configuration of an amino acid from l to d is a simple way of altering its side chain orientation (Schmidt et al. 1991). This change applied to Arg^8^ in AVP results in a selective antidiuretic agonist ([d-Arg^8^]VP, DAVP) (Lebl et al. 1987). Moreover, from the structural point of view, the d-enantiomers, having energy minima in enantio-symmetry-related areas of the Ramachandran map relative to their l-counterparts, are particularly suitable for stabilizing *β*-turns: in the (*i* + 1) position—type II’ one, and in the (*i* + 2) position—type II turn (Toniolo and Benedetti 1991). Furthermore, the change of configuration of an amino acid from native l to d increases resistance of a peptide to enzyme-catalyzed breakdown, which can dramatically increase serum half-life (Milton et al. 1992; Sadowski et al. 2004). Short d-peptides can be absorbed systemically when taken orally, whereas l-peptides must be injected to avoid digestion (Pappenheimer et al. 1994, 1997). Therefore, d-peptides are potentially attractive for drugs (Welch et al. 2007). Another method to obtain peptides more resistant to proteolytic degradation is glycosylation. However, introduction of sterically restricted bulky glycoconjugates usually significantly reduces the activity of analogues by steric repulsion between carbohydrate moieties and the receptors (Kihlberg et al. 1995; Lubecka et al. 2014).

In this project, we searched dominant conformations of antidiuretic agonists, arginine-vasopressin (AVP, Fig. 1S in supplementary material) and d-arginine-vasopressin (DAVP), in micelles and liposomes. We checked how the configuration reversal of all amino acid residues (from l to d and vice versa) in native AVP and DAVP would affect biological potency and structure of the peptides. We synthesized and analyzed AVP and DAVP, and their *inverso* analogues, *inverso*-AVP (iAVP) and *inverso*-DAVP (iDAVP). We used CD spectroscopy to analyze the influence of the liposomes and micelles on conformations of the peptides. The peptides were studied in the following liposomic media: zwitterionic DPPC, anionic DPPG and mixed DPPC/DPPG liposomes (at mole ratios 9:1 and 7:3) and micellar environment: zwitterionic DPC, anionic SDS and mixed anionic–zwitterionic micelles (mixed SDS and DPC micelles at a mole ratio 1:5). The 3D structures of the peptides were determined in the mixed anionic–zwitterionic micelles using nuclear magnetic resonance (NMR) spectroscopy supported by molecular dynamics simulations (MD). By comparison of the results obtained at different conditions, and for the active and inactive analogues we wanted to find out if and how large are differences in interactions of the active and inactive peptides with the membrane. In particular, we wanted to examine the role played by hydrophobic and electrostatic interactions between the peptides and the liposomes and to find out whether these interactions can or cannot be crucial for structures and, indirectly, also for activity of the peptides.

## Materials and methods

### Peptide synthesis

All the peptides were obtained manually by solid-phase peptide synthesis using Fmoc-chemistry on polystyrene resin (Fmoc-Gly TentaGel S RAM, Rapp Polymere, 0.23 mmol/g) on a 100-mol scale.

Mixtures of protected amino acid/2-1*H*-(benzotriazole-1-yl)-1,1,3,3-tetramethyluronium tetrafluoroborate (TBTU)/1-hydroxybenzotriazole (HOBt)/4-methylmorpholine (NMM) (1:1:1:2) in *N*,*N*-dimethylformamide (DMF) or protected amino acid/*O*-(7-azabenzotriazol-1-yl)-1,1,3,3-tetramethyluronium hexafluorophosphate (HATU)/1-hydroxy-7-azabenzotriazole (HOAt)/NMM (1:1:1:2) in DMF or in a mixture of *N,N*-dimethylformamide/1-methyl-2-pyrrolidone (NMP) (1:1 v/v) containing 1 % Triton were used for coupling. The completeness of each coupling reaction during the synthesis was monitored by chloranil test (Christensen 1979). Recoupling was performed when the test was positive.

The Fmoc deprotection was accomplished using a 20 % solution of piperidine in DMF. A solution of trifluoroacetic acid (TFA)/phenol/triisopropylsilane (TIS)/water (92.5:2.5:2.5:2.5) was used for the cleavage of the peptides from the TentaGel resin (3 h). Solutions of the cleaved peptides were filtered off and evaporated in vacuo to ca. 1 ml. The peptides were then precipitated with diethyl ether to afford crude products (about 100 mol each) which were dissolved in acetic acid (250 ml) and the solutions were diluted with methanol (1500 ml).

The resulting dithiols were oxidatively cyclized with a 0.1 M I_2_ in methanol using the standard procedure (Flouret et al. 1979). The solvents were evaporated under reduced pressure and the residue was dissolved in water and lyophilized. The crude products were desalted on a Sephadex G-15 column, and eluted with aqueous acetic acid (30 %) at a flow rate of 3 ml/h. After freeze-drying, fractions comprising the major peak were purified by RP-HPLC. The peptides were eluted as single peaks. The purity and identity of each peptide were determined by HPLC and MALDI TOF mass spectrometry (molecular ion).

### Biological evaluation

Wistar rats were used in all experiments. Handling of the experimental animals was performed under supervision of the Ethics Committee of the Academy of Sciences according to § 23 of the law of the Czech Republic No. 246/1992.

The uterotonic test was carried out in vitro in the absence of magnesium ions (Holton 1948; Munsick 1960; Slaninová 1987). Female rats were estrogenized 48 h before the experiment. The vasopressor test was performed using phenoxybenzamine-treated male rats (Dekanski 1952). Synthetic oxytocin (PolyPeptide Labs Ltd.) was used as a standard in uterotonic tests, and synthetic arginine vasopressin (PolyPeptide Labs Ltd.) was used in the pressor test. Dose–response (single administration) or cumulative dose–response (measurements without washing steps between the administration of enhanced doses) curves were constructed. The values reported are averages of three to five independent experiments.

Tests to assess the antidiuretic or diuretic properties were conducted on conscious male rats in two variations of the modified Burn test (Burn et al. 1950; Vávra et al. 1974). Antidiuretic activity was tested using hydrated rats. The animals fasted for 16 h were weighed and then given tap water through a stomach catheter. The water load was 4 % of the body weight. Immediately after the water load, the tested substances (or physiological saline as control) were administered subcutaneously at doses of 0.001–100 nmol/kg. The rats were then placed in separate metabolic cages, and their urine collected over a 5-h period. The time in which the rats excreted half the water load (*t*_1/2_) was determined and plotted against the dose. For comparison of the compounds, such doses were chosen yielding *t*_1/2_ equal to 60 min (the so-called threshold doses, equal to the value of *t*_1/2_ obtained with the physiological solution) and 200 min. On each day of the experiment, 21 rats were divided into five groups of four or five animals to which different doses of different compounds were administered. To test the diuretic effects, the nonhydrated rats were used, i.e., no water load was given to the fasting animals (for details see Slaninová 1987).

### Preparation of liposomes

Multilamellar vesicles (MLVs) consisting of liposomes were prepared by dissolving the lipids up to a concentration of 3 mg/ml in the chloroform/methanol (2:1, v:v). Subsequently, they were evaporated under argon flow and desiccated under vacuum overnight to remove any residual solvents. The lyophilized peptide was blended with the MLV suspension in distilled water under ultrasonic condition that reduces the size of liposomes. Then, the peptide–liposome mixture was incubated for 2 h at 45 °C with gentle vortex mixing. Next, the samples were frozen and thawed over five cycles to decrease lamellarity and reduce the liposomes size (du Plessis et al. 1996). A single freeze–thaw cycle consisted of freezing for 5 min at a dry ice temperature (−78 °C) and subsequent thawing for 5 min in a water bath at 45 °C.

### CD measurements

Circular dichroism spectra were collected using a Jasco J-815 spectropolarimeter (Physicochemical Laboratories, Faculty of Chemistry, University of Gdansk). Initially, the CD spectra of the peptides were recorded in distilled water and 10 mM of a pH 7.4 phosphate buffer (2 mM KH_2_PO_4_, 10 mM Na_2_HPO_4_, 137 mM NaCl, and 2.7 mM KCl). However, because of strong interference of the phosphate buffer below 200 nm, subsequent CD measurements were carried out in distilled water with addition of the DPPC, DPPC/DPPG (9:1, mol:mol), DPPC/DPPG (7:3, mol:mol) and DPPG liposomes. The concentration of the peptides was 0.14 mg/ml. The total peptide:lipid ratio was approximately 1:35. All the measurements in the liposomes were conducted over the temperature range of 25–45 °C at a 5 °C interval. The spectra were recorded separately over the ranges of 185–260 nm and 230–340 nm, with a 1- and 10-mm path length, respectively. Afterwards, the CD measurements were carried out in water with addition of the DPC, SDS or DPC/SDS (5:1, mol:mol) micelles. The concentration of the detergents was 20 mg/ml. All the measurements in the micelles were conducted on 0.1 mg/ml peptide solutions at room temperature. The background of the water, lipid or detergent solution was subtracted from the CD data. The spectra were plotted as mean molar ellipticity Θ (degree × cm^2^ × d mol^−1^) vs. wavelength *λ* (nm). The signal/noise ratio was increased by acquiring each spectrum over an average of three scans.

### NMR measurements

The peptides for the NMR measurements were dissolved in 10 mM of a pH 7.4 phosphate buffer (90 % H_2_O and 10 % D_2_O; 2 mM KH_2_PO_4_, 10 mM Na_2_HPO_4_, 137 mM NaCl, and 2.7 mM KCl) with addition of the mixed anionic–zwitterionic micelles (SDS and DPC at a mole ratio of 1:5). The deuterated detergents SDS-d_25_ and DPC-d_38_ were purchased from Sigma-Aldrich. A typical sample concentration was 4.5 mM of a peptide, 26 mM SDS-d_25_, and 130 mM DPC-d_38_. The total peptide:detergent ratio was approximately 1:35. The NMR spectra were recorded on a 500 MHz Varian spectrometer, equipped with the Performa II gradient generator unit, WFG, Ultrashims, a high stability temperature unit and a 5 mm ^1^H{^13^C/^15^N} PFG triple resonance inverse probe head, at the Intercollegiate Nuclear Magnetic Resonance Laboratory at the Gdansk University of Technology.

The 2D NMR spectra were measured at 32 °C. The temperature coefficients of the amide proton chemical shifts were established from a set of 1D ^1^H NMR spectra for the following temperatures: 5, 10, 20, 32, 40, and 50 °C. Proton resonance assignments were accomplished using the proton–proton total chemical shift correlation spectroscopy (TOCSY) (Bax and Davis 1985a), the Nuclear Overhauser effect spectroscopy (NOESY) (Kumar et al. 1980), the rotating-frame Overhauser enhancement spectroscopy (ROESY) (Bothner-By et al. 1980; Bax and Davis 1985b), as well as the gradient heteronuclear single quantum coherence spectroscopy (^1^H-^13^C gHSQC) (Palmer et al. 1991; Kay et al. 1992; Schleucher et al. 1994). For each peptide, the TOCSY spectra were recorded with a spin-lock field strength of 12.2 kHz and a mixing time of 80 ms. The mixing times of the NOESY experiments were set to 150 and 200 ms. ROESY data were collected with the mixing time of 200 ms. The volumes of cross-peaks were picked up for the NOESY spectra with a mixing time of 150 ms.

Vicinal coupling constants, ^3^*J*_HNHα_, were assigned using the double-quantum-filtered correlation spectra (DQF-COSY) (Rance et al. 1983) and the 1D NMR spectra. The DQF-COSY spectra were processed to enhance the resolution to 1.2 Hz per point in F2. The homonuclear spectra were recorded with 64 scans per *t*1, a spectral width of 5 kHz in both dimensions, and 512 × 2 K data sets, which were then zero-filled to 1 K × 2 K after Fourier transform. The two ^3^*J*_HNHα_ coupling constants with Hα protons for Gly^9^ are equal within the limits of error. Data size for the ^1^H-^13^C gHSQC spectrum was 256 (*t*1)–1024 (*t*2), and spectral widths were 18 kHz in the ^13^C dimension and 5 kHz in the ^1^H dimension. In this experiment, a total number of 128 transients were used.

The spectra were calibrated against a HDO signal frequency measured with respect to external 2,2-dimethyl-2-silapentanesulphonic acid (DSS). The ^13^C chemical shifts were referenced to DSS according to the formula: ^13^C/^1^H = 0.251449530 (Wishart et al. 1995). Spectral processing was carried out using VNMR 6.1B (Varian Inc., Palo Alto, CA, USA), and analyzed with SPARKY tool (Goddard and Kneller 2008).

### Molecular modeling

Molecular dynamics (MD) simulations were carried out with the parm99 force field using the AMBER 11 package (Case et al. 2010). The valence geometry of the d-amino acids, not specified in the standard AMBER database, was parameterized as recommended in the AMBER 11 manual. Initial structures of the peptides were generated in random conformation. Each peptide was placed in a simulation box together with ten monomers of sodium dodecyl sulphate and 50 monomers of dodecylphosphocholine using the PACKMOL programme (Martínez and Martínez 2003; Martínez et al. 2009). A single SDS molecule has previously been modeled and parameterized (Rodziewicz-Motowidło et al. 2008). The geometry of the single molecule of DPC was taken from a pre-built hydrated DPC micelle (Tieleman et al. 2000). The DPC topology was adapted to the AMBER 11. The initial solvent configuration around all molecules was obtained by filling a cubic box with water molecules. About 13,500 water molecules were added. The sodium ions were added to neutralize the entire system. The overall box size was about 85 Å in each direction, which corresponded to the concentrations of SDS and DPC equal to 26 and 130 mM, respectively, coinciding with that of the NMR experiment. Finally, a simulation under a fixed pressure at 301 K in the periodic box was carried out to equilibrate the entire system. After 60–80-ns simulations we obtained a stable anionic–zwitterionic micelle/peptide/water system, with the peptide on micelle surface (see Fig. 2S in supplementary material).

After equilibration of the system, a time-averaged (TAV) restraints method (Torda et al. 1989) was used and the restraints imposed on interproton distances and dihedral angles determined from NOE intensities and coupling constants, respectively. TAV provides a better approximation of the physical nature of the NOE, assuming that NOEs’ cross-peaks represent averages of multiple conformations in solution (Torda et al. 1989). MD simulations with TAV (TAV-MD) were carried out at 301 K in the NPT ensemble during 8 ns. The time step was 2 fs. The coordinates were collected every 2,000th step. The interproton distances were restrained with the force constants *f* = 20 kcal/(mol × rad^2^) and the dihedral angles with *f* = 2 kcal/(mol × rad^2^). The geometry of the peptide groups (all *trans*) was kept fixed according to the NMR data by imposing *f* = 50 kcal/(mol × rad^2^) on the *ω* torsion. The conformations obtained during the last 1 ns of simulation were considered in further analysis. As a result, the set of 250 conformations for each peptide was obtained.

A total of 75, 73, 69, and 92 NOEs were assigned from the NOESY spectra of AVP, iAVP, DAVP, and iDAVP, respectively. The interproton distances used in TAV-MD were calculated on the basis of NOEs intensities by the CALIBA algorithm in the DYANA (Güntert et al. 1997) programme. The backbone ^3^*J*_HNHα_ coupling constants were converted to backbone torsion angle *φ* constraints according to the following rules: ^3^*J*_HNHα_ < 6 Hz constrained the *φ* angle to the range of −90° to −30°, 6 Hz < ^3^*J*_HNHα_ < 8 Hz constrained it to −120° to −60°, 8 Hz < ^3^*J*_HNHα_ < 9 Hz constrained *φ* to −160° to −80° and ^3^*J*_HNHα_ > 9 Hz constrained it to −140° to −100° (Williamson et al. 1985; Pardi et al. 1984; Eberstadt et al. 1995). With d-amino acids, their allowed regions in Ramachandran map are inverted in both *φ* and *ψ* when compared to the values for L amino acids (*φ*, *ψ* → −*φ* and −*ψ*) (Ramachandran et al. 1963). Therefore, the constrains for d-amino acids were rebuilt to suit them.

The results were analyzed using the Carnal and Ptraj programmes from the AMBER 11 package (Case et al. 2010). Two measures of mutual relations between the aromatic rings of tyrosine 2 and phenylalanine 3 were introduced: (1) the distance between the centers of mass of the aromatic rings (*Dis*_Tyr–Phe_), and (2) the dihedral angle determined by four points: (a) the center of mass of the aromatic ring of Tyr^2^, (b) Cα of Tyr^2^, (c) Cα of Phe^3^ and (d) the center of mass of the aromatic ring of Phe^3^ (*Ang*_Tyr–Phe_). The mass centers were defined by three carbon atoms from aromatic rings, C_1_, C_3_, C_5_ (IUPAC) for both residues. To find the peptide–micelle interactions, the radial distribution functions (RDFs) between the side chains and head groups of the mixed anionic–zwitterionic micelle and also between the side chains and the micelle’s core were calculated. The data were averaged over the final conformations. To estimate the interactions between the peptides and aqueous environment, hydration numbers were also calculated, as a method of quantifying the interactions between the peptides and water. The hydration number is an integral radial distribution function showing how many water molecules are located near the residue. Two sets of the hydration numbers were calculated for each system, one for the side chains heavy atoms, and the other for the carbonyl oxygen for each residue for a hydration radius of 3.8 Å.

The molecular structures were drawn and analyzed with the graphic programmes RASMOL (Sayle and Milner-White 1995) and MOLMOL (Koradi et al. 1996).

## Results and discussion

### Biological activities

Pharmacological properties of the peptides studied are collected in Table 1S (in supplementary material). AVP displays antidiuretic, pressor and oxytocic activities (Lebl et al. 1987) and DAVP is a selective antidiuretic agonist (Lebl et al. 1987; Slaninová 1987). In turn, our results have shown that the *inverso* analogues of AVP and DAVP are inactive against renal (V2, antidiuretic), vascular (V1a, pressor), and oxytocic (OT) receptors.

### Analysis of the CD spectra in water

**Fig. 1:**
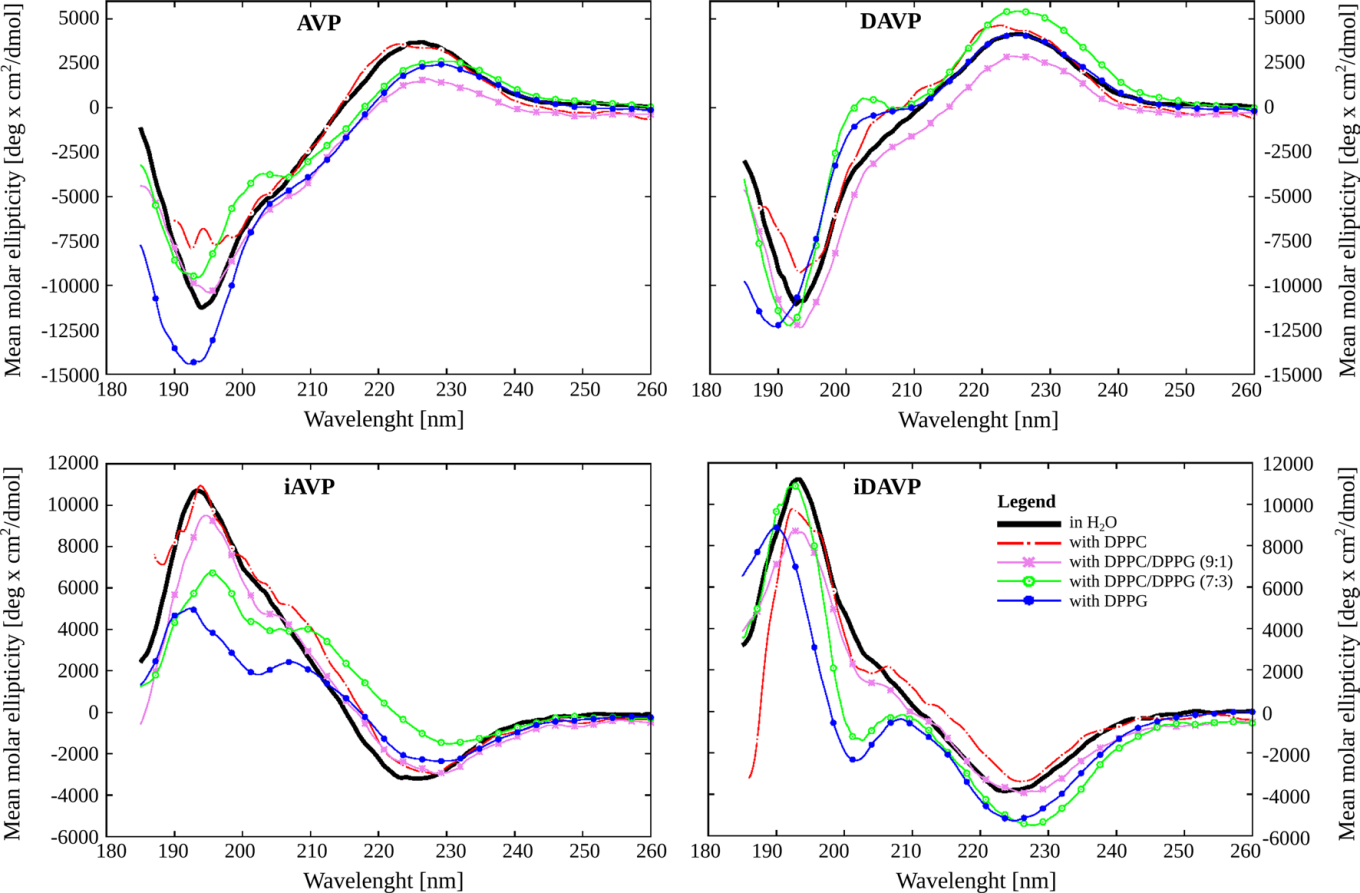
The liposome dependence of the far-UV CD spectra of the peptides at 25 °C, where *AVP* arginine-vasopressin, *iAVP* *inverso*-AVP, *DAVP* [d-Arg^8^]-AVP and *iDAVP* *inverso*-DAVP

The far-UV CD spectra of the peptides were studied in various solvents. In the lipid-free aqueous solution the CD spectra of AVP and DAVP show similar number and positions of the extremes over the range 185–260 nm (Fig. 1). The 200 nm region includes a pair of negative bands, at 205 and 195 nm. The bands are contributed by π–π* transition of the amide groups, and the shorter-wavelength one is also due to the E1u transition of the aromatic ring of Tyr^2^. In both cases the B1u band of Phe^3^ transition, usually emerging around 217 nm, was missing. It was apparently hidden by the more intense bands of other transitions appearing in the same spectral region (Fric et al. 1975). The CD spectra of the *inverso* analogues, iAVP and iDAVP, have the same number and positions of the extremes as their respective parent peptides, but the negative extremes in *inverso* analogue spectrum correspond to the positive extremes in AVP or DAVP, and vice versa. Thus, in the nonchiral solvent such as water, the spectra are mirrored between pairs of the respective enantiomers. The temperature has only a slight influence on conformation of the peptides in the lipid-free solution (Fig. 3S in supplementary material). The same relationships hold in the 230–340-nm range (Fig. 4S in supplementary material).

### Analysis of the CD spectra in the liposomes

Addition of the liposomes to the water changed the shape of the CD spectra of the peptides in the 185–260-nm range (Fig. 1) and it differentiated the shapes of the spectra of AVP/DAVP and their *inverso* analogues. This indicates that all the peptides interact with the liposomes and these interactions are different for different peptides. The smallest differences were observed for DPPC and the largest for DPPG (Fig. 1). The shape of the CD spectrum in DPPC is usually similar to that in water, but the band intensities are different. This suggests some conformational changes to have occurred. Again, in the presence of the DPPG liposomes the band around 205 nm is noticeably stronger than that in water or DPPC liposomes, where in this region only a small shoulder is seen. This reflects the presence of more ordered structures of the peptides in liposomes. A similar effect is observed for the mixed DPPC/DPPG (9:1) and (7:3) liposomes, but for the DPPC/DPPG (9:1) the intensity of the 205-nm band is not so strong.

The complex 227-nm band assigned to superposition of the positive band of the amide *n*–π* transition and the aromatic B1u transition of tyrosine (Corrêa and Ramos 2009), with AVP is weaker in the presence of DPPG and the mixed DPPC/DPPG liposomes, and is slightly red-shifted in 25 °C [from 226.4 nm in water to 227.2, 228.8 and 229.4 nm in DPPC/DPPG (9:1), DPPC/DPPG (7:3) and DPPG, respectively (Fig. 1)]. In turn, with DAVP the 227-nm band is noticeably stronger in DPPG and the DPPC/DPPG (7:3) liposomes than in water. This band in DAVP is also distinctly stronger than in AVP, and it emerges at a slightly lower wavelength. This band, for *inverso*-analogues in the DPPG and DPPC/DPPG (7:3) liposomes, appears at a higher wavelength, and it is less intense for iAVP and more intense for iDAVP than in water. At the same time, in the DPPC and DPPC/DPPG (9:1) liposomes, the position and intensity of this band are similar to those in lipid-free environment. The strong 227-nm band of vasopressin, as previous studies have indicated, is due to mutual arrangement of the aromatic side chains of Tyr^2^ and Phe^3^, being extended away of the macrocyclic ring and stacking face to face in aqueous solution (Fric et al. 1975; Tu et al. 1979), as confirmed by NMR studies (Sikorska and Rodziewicz-Motowidło 2008). The π–π interactions certainly restrict conformational freedom of the Tyr^2^ side chain and stabilize its proper orientation, crucial for antidiuretic activity (Walter 1977). Thus, the differences in intensity of the 227-nm band suggest that specific interactions of the aromatic nuclei of tyrosine and phenylalanine for the AVP and iAVP are weakened upon binding to the DPPG and mixed DPPC/DPPG liposomes, this being consistent with AVP-data reported recently (Sikorska et al. 2012). Moreover, the strong 227-nm band of DAVP may explain a potent antidiuretic activity of this peptide.

**Fig. 2:**
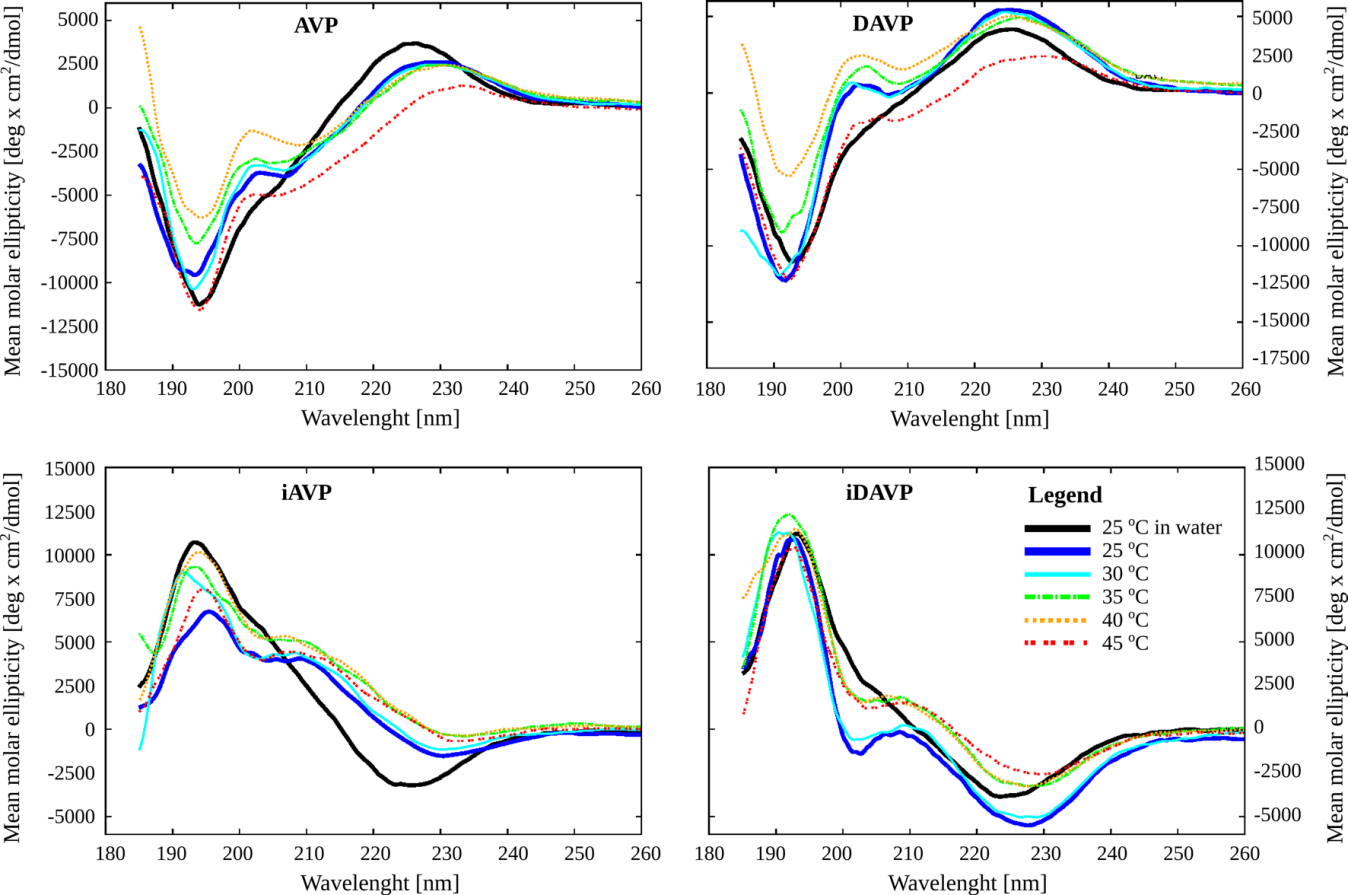
The temperature dependence of the far-UV CD spectra of the peptides in the presence of DPPC/DPPG (7:3, mol:mol) liposomes, where *AVP* arginine-vasopressin, *iAVP* *inverso*-AVP, *DAVP* [d-Arg^8^]-AVP and *iDAVP* *inverso*-DAVP

In the presence of the DPPG (Fig. 7S in supplementary material) and mixed DPPC/DPPG liposomes (Fig. 2 and Fig. 6S in supplementary material), the temperature affects the shape of the CD spectra of the peptides, while in the DPPC liposomes this effect is only slight (Fig. 5S in supplementary material). In the DPPC/DPPG (9:1) liposomes for all the peptides the complex band at 227 nm decreases in magnitude upon raising the temperature, which is consistent with AVP-data reported recently (Sikorska et al. 2012). In turn, in the DPPG liposomes, upon raising the temperature, a slight gradual increase in intensity of the band is noticed, and in the DPPC/DPPG (7:3) liposomes the intensity of the 227-nm band increases over the range of 25–40 °C and then decreases at 45 °C, i.e., above the temperature of main phase transition (*T*m). The decrease is strong at 45 °C for AVP and DAVP. The changes in intensity of the 227-nm band in the DPPC/DPPG (7:3) liposomes suggest that in the gel phase of the lipids the Tyr–Phe aromatic interactions are stronger than in the liquid crystalline phase. Thus, the temperature has an impact on conformation of the peptides in lipid solutions, as well as on the peptides–liposomes interactions. In the zwitterionic DPPC liposomes, below Tm, most of all, hydrophobic interactions with the peptides can be expected (Thakur et al. 2014). Above *T*m, in the liquid crystalline phase, the negatively charged phosphatidyl groups are more exposed (Chen and Tripp 2012), whereas in the DPPG and mixed DPPC/DPPG liposomes, also hydrophobic interactions occur, but the presence of anionic DPPG adds up negative charges resulting in additional attractive electrostatic interactions even below *T*m.

In the 230–340-nm range, in the presence of the DPPC/DPPG (7:3) liposomes, the negative band around 280 nm is more intense for AVP and DAVP, and upon raising the temperature, it decreases in magnitude (Fig. 8S in supplementary material). This band is due to a combination of the disulphide *n*–σ* and tyrosine low energy π–π* transitions (Hruby et al. 1982). In the long-wavelength CD spectra of the neurohypophyseal hormones around 250 nm also a positive shoulder or band assigned to the disulphide *n*–σ* transitions emerges occurs (Tu et al. 1979; Urry et al. 1968). This band is very intense for oxytocin in the lipid-free solution and decreases sharply upon binding to liposomes, whereas with vasopressin only a slight inflection in the CD curve in water is seen, which disappears altogether in the liposome solution (Sikorska et al. 2012). Our results suggest that the last tendency seems to be typical of vasopressin-like peptides—we have found the same relationship for both AVP and DAVP. In turn, for iAVP there are no differences in the shape of CD spectra between the solution with or without lipids, and for iDAVP the 255-nm shoulder is more intense upon binding to the liposomes (below 40 °C) than in water.

In summary, the presence of liposomes differentiated the shapes of the CD spectra of vasopressin-like peptides. The smallest differences occurred with zwitterionic DPPC, the largest—with anionic DPPG and intermediate ones—with mixed anionic–zwitterionic liposomes.

### Analysis of the CD spectra in the micelles

**Fig. 3:**
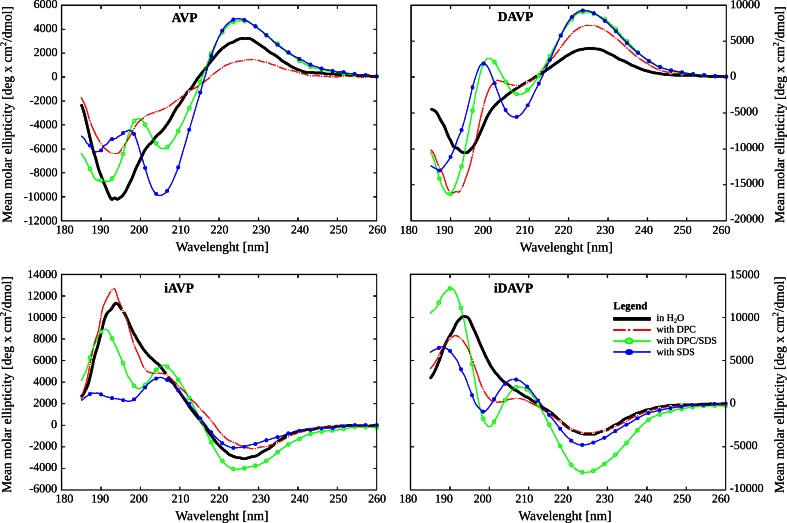
The micelle dependence of the far-UV CD spectra of the peptides at 25 °C, where *AVP* arginine-vasopressin, *iAVP* *inverso*-AVP, *DAVP* [d-Arg^8^]-AVP and *iDAVP* *inverso*-DAVP

As already mentioned, DPC and SDS micelles are currently the most commonly used “membrane mimics” for studies of peptide–membrane interactions (Beswick et al. 1998a, b; Strandberg and Ulrich 2004; MacKenzie et al. 1997; Pages et al. 2009). For this reason, CD spectroscopy was also used to analyze conformational changes in the peptides upon binding to the SDS, DPC and mixed DPC/SDS (5:1) micelles. Addition of the micelles to the water also differentiated the shapes of the CD spectra of AVP/DAVP and their *inverso* analogues (Fig. 3). The smallest differences in the shape of the CD spectra were observed with the zwitterionic DPC micelle. The same tendency was noticed in the presence of the zwitterionic DPPC liposomes. The shapes of the CD spectra usually do not differ much from those in water, but some differences in band intensities are observed. In turn, in the SDS solution, the 205-nm band is noticeably stronger than in water or in the DPC micelle. In this region, only a small shoulder is noticed for the DPC micelle. Moreover, the 205-nm band in the spectra of the AVP and iAVP, is even stronger than the 195-nm one, which reflects the presence of more ordered structures. In the mixed DPC/SDS micelle a similar effect is observed, but the intensity of the 205-nm band is not so strong.

The shapes of the CD spectra of the peptides in the mixed DPC/SDS (5:1) micelles (Fig. 3) are usually similar to those in the DPPC/DPPG (7:3) liposomes (Figs. 1, 2). They are only slightly different in intensities and band positions. Bearing this in mind, and the fact that detergents, such as DPC and SDS, are more useful than liposomes in NMR structural studies in solution (Warschawski et al. 2011), we decided to use the mixed DPC/SDS micelles as an environment for the NMR measurements.

### Analysis of the NMR spectra

**Fig. 4:**
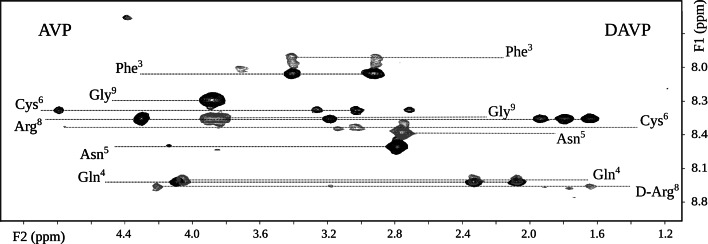
The fingerprint regions of the TOCSY spectra of AVP (*black*) and DAVP (*gray*) showing the correlation between the amide protons and the side-chain protons

**Fig. 5:**
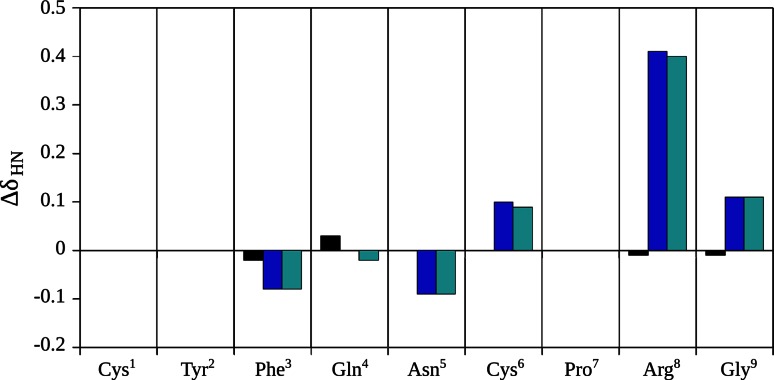
The differences between the amide proton chemical shifts of native vasopressin (AVP) and those of the *inverso*-AVP (*black*), DAVP (*blue*) and *inverso*-DAVP (*cyan*), (Δ*δ*_NH_ = *δ*_analogue_ − *δ*_AVP_)

We adopted the conventional strategy for interpretation of the NMR spectra (Wüthrich 1986). The chemical shifts of most protons of *inverso*-AVP are very similar to those of the parent hormone. A similar relationship holds for DAVP and its *inverso* analogue (Table 1). However, the differences between epimeric ^1^HN chemical shifts in Arg^8^ in the AVP–DAVP and iAVP–iDAVP pairs are considerable. The fingerprint region of the TOCSY spectra of AVP and DAVP is shown in Fig. 4. The differences between the backbone amide proton chemical shifts of native AVP and its analogues (iAVP, DAVP and iDAVP) are shown in Fig. 5.

**Table 1:** Proton chemical shifts (ppm) of AVP, *inverso*-AVP, DAVP and *inverso*-DAVP in a pH 7.4 phosphate buffer (90 % H_2_O and 10 % D_2_O) in the presence of the mixed anionic–zwitterionic micelles at 32 °C

Residue	Proton type	Chemical shifts (ppm)			
		AVP	*inverso*-AVP	DAVP	*inverso*-DAVP
l-Cys^1^/d-Cys^1^	HN	No	No	No	No
	Hα	3.90	3.88	3.91	3.88
	Hβ	2.99, 3.47	2.96, 3.47	3.01, 3.56	2.99, 3.55
l-Tyr^2^/d-Tyr^2^	HN	No	No	No	No
	Hα	4.24	4.23	4.16	4.16
	Hβ	2.65	2.65	2.61, 2.70	2.61, 2.69
	H2,5	6.75	6.74	6.65	6.65
	H3,4	6.65	6.65	6.62	6.62
l-Phe^3^/d-Phe^3^	HN	8.03	8.01	7.95 (7.92)	7.95
	Hα	No	No	No	4.63
	Hβ	2.91, 3.40	2.90, 3.41	2.90, 3.40 (2.88, 3.41)	2.89, 3.40
	H2,6	7.40	7.40	7.41	7.40
	H3,5	7.28	7.27	7.28	7.27
	H4	7.15	7.15	7.14	7.14
l-Gln^4^/d-Gln^4^	HN	8.68	8.71	8.68 (8.66)	8.66
	Hα	4.08	4.07	4.05 (4.07)	4.05
	Hβ	2.07	2.07	2.07 (2.08)	2.06
	Hγ	2.32	2.32	2.32 (2.32)	2.32
	ε-NH2	6.79, 7.57	6.79, 7.57	6.79, 7.57	6.79, 7.57
l-Asn^5^/d-Asn^5^	HN	8.47	8.47	8.38 (8.33)	8.38
	Hα	4.64	No	No	No
	Hβ	2.78	2.78	2.76 (2.74)	2.76
	δ-NH2	6.81, 7.65	6.81, 7.65	6.83, 7.66	6.83, 7.66
l-Cys^6^/d-Cys^6^	HN	8.26	8.26	8.36	8.35
	Hα	4.78	4.78	4.76	4.75
	Hβ	3.02, 3.25	3.03, 3.27	3.01, 3.14	3.01, 3.14
l-Pro^7^/d-Pro^7^	Hα	4.38	4.37	4.44	4.44
	Hβ	1.89, 2.25	1.89, 2.25	1.85, 2.24	1.85, 2.24
	Hγ	1.98	1.98	1.93, 2.03	1.93, 2.03
	Hδ	3.67, 3.87	3.66, 3.89	3.74, 3.79	3.74, 3.79
l-Arg^8^/d-Arg^8^	HN	8.30	8.29	8.71	8.70
	Hα	4.29	4.30	4.20	4.20
	Hβ	1.78, 1.92	1.78, 1.93	1.77, 1.90	1.77, 1.90
	Hγ	1.63	1.64	1.63	1.63
	Hδ	3.17	3.18	3.17	3.17
	ε-NH	8.93	8.93	No.	No.
Gly^9^	HN	8.19	8.18	8.30	8.30
	Hα	3.88, 3.91	3.88	3.83, 3.89	3.83, 3.89
C-NH_2_	NH2	No	No	No	No

Values in parentheses are chemical shifts for the minor species

*No* not observed

The NMR spectra of DAVP indicate that residues Phe^3^, Gln^4^ and Asn^5^ exist at equilibrium between two conformational states (Fig. 4). The intensity ratio of both conformers is equal approximately to 1.5:1. The remaining residues have only one set of chemical shifts. Appearance of two conformers may be the result of either the *cis/trans* isomerization of the Cys^6^–Pro^7^ peptide bond (Larive et al. 1992; Larive and Rabenstein 1993; Walse et al. 1998; Lubecka et al. 2012), and/or a slow, on the NMR time scale, mobility of these fragments and/or diverse interactions of the peptides with micelle. The *cis* and *trans* conformations of the X-proline peptide bond are often similar in energy and may both be populated under physiological conditions. The first example of the *cis*–*trans* isomerism of the X-proline peptide bond in nonoligomeric short peptides was reported for the *S*-benzyl-Cys-Pro-Leu-Gly-NH_2_ (the acyclic part of oxytocin). Resonances for both isomers were observed in ^1^H NMR spectra of the DMSO solutions of this peptide, at a *trans* to *cis* ratio of about 3–2 (Hruby et al. 1971). In turn, in aqueous solution, the proportion share is 10 % for the *cis* isomer of oxytocin (Larive et al. 1992) and 5–9 % for vasopressin (Sikorska and Rodziewicz-Motowidło 2008; Larive et al. 1992). The *cis/trans* isomerization hypothesis could be verified by an exchange *trans*Hα(Pro)–*cis*Hα(Pro) cross-peak and/or *cis*Hα(Cys)–*cis*Hα(Pro) connectivity. Neither was found in the ROESY and/or NOESY spectra, because of the wide signal of water, exactly between the signals of Hα of Cys^6^ and Hα of Pro^7^. The ROESY and/or NOESY spectra taken at a higher temperature did not solve the issue. Additionally, we have not observed the resonances for the *cis* isomer in the ^1^H-^13^C NMR spectra of the peptides. The *cis* isomer was also missing in the ^13^C NMR spectra of oxytocin in DMSO or in other solvents (Brewster et al. 1973), and of vasopressin in aqueous solution (Larive et al. 1992).

Only one set of proton resonances seen for the positively charged fragments of the peptide, that is for Cys^1^ and d-Arg^8^, may be due to electrostatic interactions with negatively charged sulphate and phosphate groups at the micelle surface, which could restrict conformational space accessible for them. However, one cannot exclude a hypothesis that the Phe^3^–Asn^4^ part of the peptide is either flexible or interacts with the micelle in different ways. To sum up, we cannot authenticate the origin of two conformers unambiguously.

**Fig. 6:**
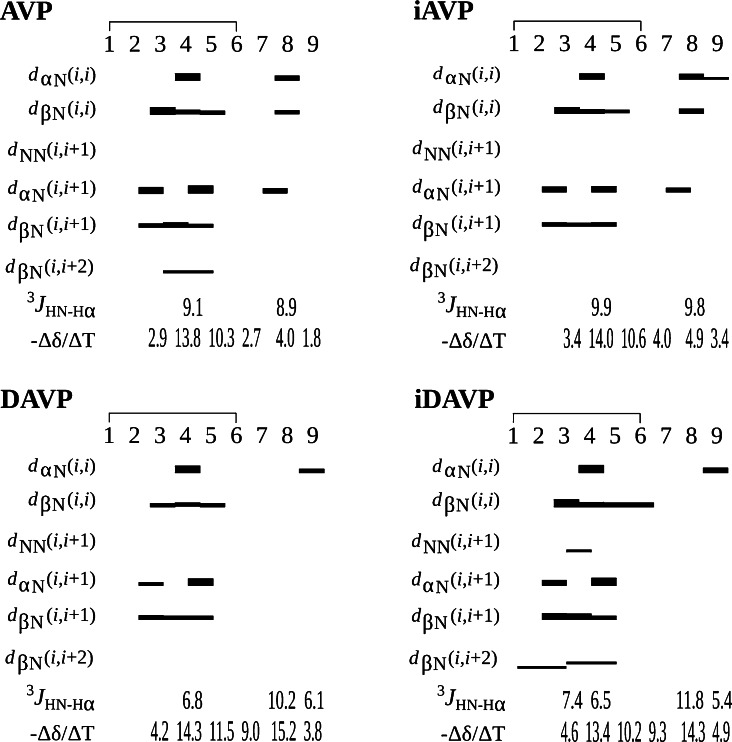
The NOE effects with their thickness indicating respective interproton distances, ^3^ *J*_HNHα_ coupling constants and the temperature coefficients of the backbone amide atoms of AVP, *inverso*-AVP (iAVP), DAVP and *inverso*-DAVP (iDAVP)

All peptide bonds of AVP, iAVP, iDAVP and the major isomer of DAVP, are in *trans* configuration, which is verified by the *d*_αN_(*i*,*i* + 1) and *d*_αδ_(*i*,*i* + 1) connectivities (Fig. 6). Moreover, small chemical shift difference between proline Cβ and Cγ (Δδ_Cβ−Cγ_ = 4.2–6.4 ppm) (Table 2) confirms the *trans* form of the Cys^6^–Pro^7^ peptide bond (Dorman and Bovey 1973).

**Table 2:** Carbon chemical shifts (ppm) of AVP, *inverso*-AVP, DAVP and *inverso*-DAVP in a pH 7.4 phosphate buffer (90 % H_2_O and 10 % D_2_O) in the presence of the mixed anionic–zwitterionic micelles at 32 °C

Residue	Carbon type	Chemical shifts (ppm)			
		AVP	*inverso*-AVP	DAVP	*inverso*-DAVP
l-Cys^1^/d-Cys^1^	Cα	53.55	53.73	53.57	53.40
	Cβ	42.40	No	42.63	42.71
l-Tyr^2^/d-Tyr^2^	Cα	57.60	57.83	No	57.68
	Cβ	37.08	36.94	36.58	36.51
	C2,5	130.28	130.34	130.04	129.99
	C3,4	115.58	115.56	115.24	115.24
l-Phe^3^/d-Phe^3^	Cα	No	No	No	No
	Cβ	37.36	37.35	No	37.20
	C2,6	129.85	129.84	129.52	129.49
	C3,5	128.64	128.67	128.17	128.22
	C4	126.83	126.62	126.29	126.35
l-Gln^4^/d-Gln^4^	Cα	55.04	55.17	54.96	54.90
	Cβ	26.66	26.68	26.49	26.48
	Cγ	31.57	31.63	31.30	31.29
l-Asn^5^/d-Asn^5^	Cα	No	No	No	No
	Cβ	35.80	35.82	35.86	35.87
l-Cys^6^/d-Cys^6^	Cα	No	No	No	No
	Cβ	39.43	39.34	No	38.96
l-Pro^7^/d-Pro^7^	Cα	61.46	61.53	No	60.89
	Cβ	29.46	29.50	29.38	29.20
	Cγ	25.17	25.22	No	24.92
	Cδ	48.26	48.29	48.00	48.03
l-Arg^8^/d-Arg^8^	Cα	53.71	53.77	54.10	54.06
	Cβ	27.98	28.15	27.86	27.90
	Cγ	24.94	24.89	24.90	24.92
	Cδ	40.98	41.01	40.91	40.88
Gly^9^	Cα	42.74	42.73	42.45	42.45

*N* *o* not observed

The temperature coefficients of the amide protons (Fig. 6) of Phe^3^, Cys^6^ and Gly^9^ of AVP fell in the range −3 < Δ*δ*/Δ*T* < 0 ppb/K, indicating occurrence of strong intramolecular hydrogen bonds. In turn, the temperature coefficients in the range −5 < Δ*δ*/Δ*T* < −3 ppb/K, indicating weak intramolecular and/or solute–solvent hydrogen bonds (Andersen et al. 1997; Baxter and Williamson 1997), were observed for HN of Phe/D-Phe^3^ and Gly^9^ of iAVP, DAVP and iDAVP, HN of Arg/d-Arg^8^ of AVP and *inverso*-AVP, and additionally HN of D-Cys^6^ of iAVP. It should be emphasized that shielding of the amide protons from exchange with the solvent might likewise arise from both peptide–micelle and intramolecular interactions (De Luca et al. 2003). The temperature coefficients of the remaining amide protons were below −5 ppb/K, which excluded stable hydrogen bonds. The analysis of the NOE effects, in relation to their interproton distances, enables to determine conformational preferences of the peptides (Dyson and Wright 1991). For each peptide, the connectivities on the NOESY spectra suggest *β*-turns in positions 2, 3; 3, 4 and/or 4, 5 (Fig. 6). Moreover, the low NH(Cys^6^) temperature coefficient in AVP (−2.7 ppb⁄K) (Fig. 6) suggests the strong hydrogen bond NH^6^–CO^3^, which may stabilize the 4, 5 *β*-turn.

The inversion of Arg^8^ configuration from l to d in AVP and from d to l in iAVP, results in an increase of the ^3^*J*_HNHα_ coupling constants of the arginine residue (from 8.9 to 10.2, and 9.8 to 11.8, respectively) and in a decrease in the ^3^*J*_HNHα_ coupling constants of the Gln^4^ from 9.1 to 6.8, and 9.9 to 6.5, respectively (Fig. 6). *Inverso* modification increases the ^3^*J*_HNHα_ coupling constants of Arg^8^. The high value of the ^3^*J*_HNHα_ coupling constant for d-Arg^8^ of DAVP and iAVP (10.2 and 9.8 Hz, respectively) corresponds to the peptide torsion angle *φ* in the range of 100°–140°, and for l-Arg^8^ of iDAVP (11.8 Hz) corresponds to *φ* in the range of −140° to −100° (Pardi et al. 1984; Eberstadt et al. 1995). In turn, the l-proline *φ* is fixed at about −60° and the proline ring has also a significant effect on *ψ* dihedral angle resulting in two minima, −40° and 150° (Williamson 1994). In d-proline *φ* and *ψ* have the same allowed values like in l-proline, but with opposite sign. Taking all these findings together, we can expect one of the four types of *β*-turns for iAVP *β*I′: *φ* (*i* + 1) = 60, *ψ* (*i* + 1) = 30, *φ* (*i* + 2) = 90, *ψ* (*i* + 2) = 0; for DAVP *β*II: *φ* (*i* + 1) = −60, *ψ* (*i* + 1) = 120, *φ* (*i* + 2) = 80, *ψ* (*i* + 2) = 0; for iDAVP *β*II′: *φ* (*i* + 1) = 60, *ψ* (*i* + 1) = −120, *φ* (*i* + 2) = −80, *ψ* (*i* + 2) = 0; and *β*VIII′: *φ* (*i* + 1) = 60, *ψ* (*i* + 1) = 30, *φ* (*i* + 2) = 120, *ψ* (*i* + 2) = −120) (Lewis et al. 1973) in the 6–9 fragment.

In summary, the inversion of Arg^8^ configuration considerably affects the NMR spectra of vasopressin-like peptides, in contrast to their *inverso* modifications.

### Conformational analysis of the calculated structures

The molecular dynamics simulations started from randomly distributed molecules of DPC and SDS at a mole ratio of 5:1 around the peptide. During the MD simulations, a spontaneous formation of mixed micelles was observed. Equilibration of the systems was monitored through the potential energy and the radius of gyration, as well as by visual inspection (Fig. 2S in supplementary material). The peptides interacted with the detergents’ chains during the whole MD simulations. The set of the last 250 conformations for each peptide from the final/stable anionic–zwitterionic micelle/peptide/water system was analyzed.

NMR conformational studies in the anionic–zwitterionic micelle confirmed literature data that 3,4 and 4,5 *β*-turns are typical of AVP. We have found type II or VII and I′ or IV *β*-turns, respectively, in these positions (Table 3). Moreover, these *β*-bends turned out to be very stable in the MD simulations. About 70 % of AVP conformations show also the tendency to create a *β*I-turn in position 6,7. DAVP, the antidiuretic agonist and a weak pressor and uterotonic agonist, exhibits a strong tendency to adopt both *β*-turns characteristic of vasopressin-like peptides: in position 3,4 (type I or III) and in position 4,5 (type I or IV).

**Table 3:** Structural statistics for the set of the final conformations of AVP, *inverso*-AVP, DAVP and *inverso*-DAVP obtained in the last 1 ns of MD simulations with time-averaged distance restraints (TAV)

Peptide statistic	AVP	*inverso*-AVP	DAVP	*inverso*-DAVP
Atomic r.m.s. differences (Å)				
Backbone atoms (1–6)	0.20	0.16	0.25	0.18
Heavy atoms (1–6)	0.54	0.44	0.54	0.50
Conformational properties				
Dominant reverse structures	3, 4 *β* II or VII	4, 5 *β* IV	3, 4 *β* I or III	4, 5 *β* IV
	4, 5 *β* I′ or IV	7, 8 *β* II′	4, 5 *β* I or IV	7, 8 *β* II′
	6, 7 *β* I^a^			
Most popular hydrogen bonds	HN^6^–CO^2^	HN^9^–CO^6^	HN^5^–CO^2^	
	HN^8^–CO^6^	HN^C-NH2^–CO^7^	HN^6^–CO^2^	
	s.c.^4^–CO^3^		HN^4^–s.c.^4^	
Radius of gyration (Å)				
Entire molecule	6.1	6.8	6.9	7.0
Heavy atoms	5.8	6.4	6.5	6.7
Heavy atoms (1–6)	4.7	5.1	4.7	5.1

^a^Occurs in approximately 70 % of the conformation

A common conformational feature of both *inverso*-analogues iAVP and iDAVP is their high propensity to form the *β*IV-turn in position 4,5 and *β*II′-turn in position 7,8. In iAVP, the *β*-turn in the C-terminal part is stabilized by the HN^9^–CO^6^ hydrogen bond (Table 3). The *β*-turns in the cyclic part of AVP and DAVP are stabilized by hydrogen bonds, whereas those in *inverso* analogues are not. The averaged radii of gyration (Rg) (Table 3) calculated using the heavy atoms of the cyclic parts are higher in the *inverso*-analogues suggesting their tocin rings to be more extended. Besides, the acyclic fragment of native AVP is noticeably less extended than that of the remaining peptides.

**Fig. 7:**
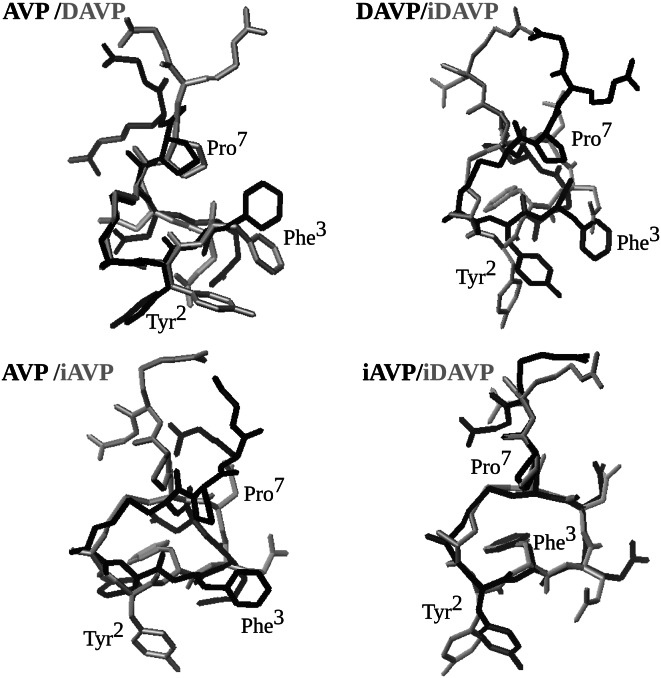
The final structures of AVP, *inverso*-AVP (iAVP), DAVP and *inverso*-DAVP (iDAVP), obtained using MD simulations with the time-averaged restraints from NMR experiments, as pictured by using MOLMOL (Koradi et al. 1996)

We also studied relative orientations of the aromatic rings, crucial for activities of the neurohypophyseal hormones. The side chains of Tyr^2^ and Phe^3^ of AVP, iAVP and iDAVP are situated away of each other and on opposite sides of the backbone (Fig. 7). The Tyr^2^ and Phe^3^ side chains of DAVP are on the same side of the tocin ring, but they are not stabilized through a π–π stacking interactions with each other. Previous studies have shown that the change of environment from water to SDS micelle considerably affects the location of the side chains and flexibility of neurohypophyseal hormones. In aqueous solution, the Tyr^2^ and Phe^3^ aromatic rings of AVP are stacked face to face to reduce the contact of the hydrophobic side chains with aqueous phase (Sikorska and Rodziewicz-Motowidło 2008), whereas in the SDS micelle, they are situated away of each other and are immersed into the hydrophobic part of the micelle (Rodziewicz-Motowidło et al. 2008). We observed similar effects in the mixed anionic–zwitterionic micelle.

**Fig. 8:**
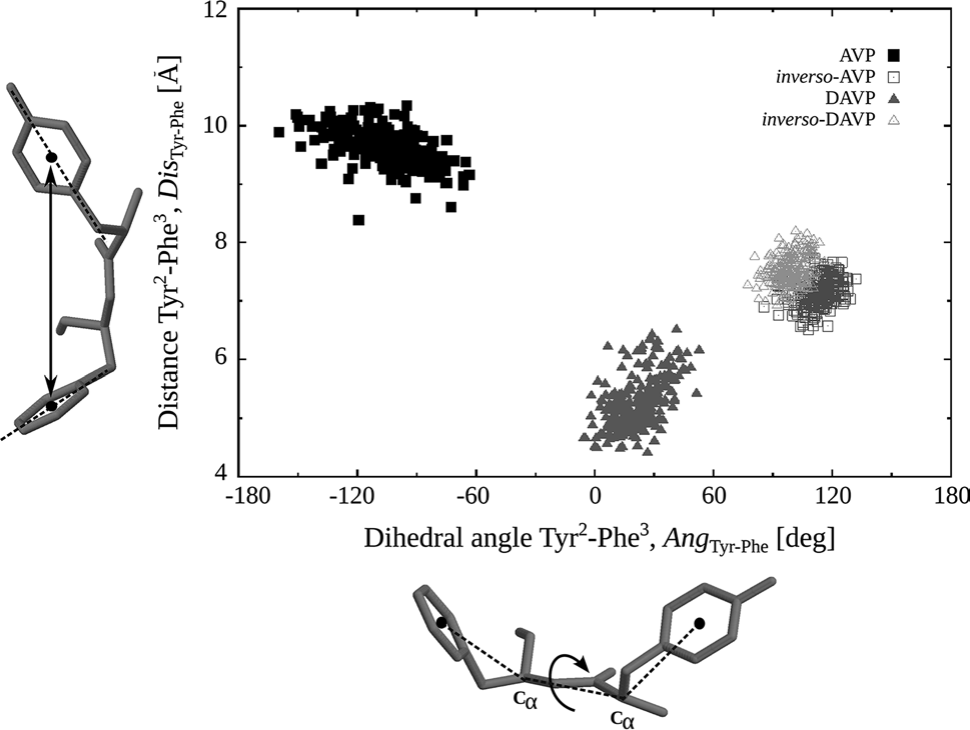
Distribution plot of the distance between the centers of mass of the aromatic rings of Tyr^2^ and Phe^3^, *Dis*_Tyr–Phe_ against the dihedral angle between two planes, where the first one is determined by the center of mass of aromatic ring of Tyr^2^, Cα of Tyr^2^ and Cα of Phe^3^, and the other determined by Cα of Tyr^2^, Cα of Phe^3^ and the center of mass of aromatic ring of Phe^3^, *Ang*_Tyr–Phe_. The mass centers were defined by three carbon atoms from aromatic rings, C_1_, C_3_, C_5_ (IUPAC)

Mutual arrangement of the Tyr^2^ and Phe^3^ aromatic rings are analyzed in greater detail using the distance (*Dis*_Tyr–Phe_), and the dihedral angle (*Ang*_Tyr–Phe_) (see Fig. 8 and “Molecular modeling”). Regarding the distance, *Dis*_Tyr–Phe_, the highest values are seen for AVP (9–10 Å) and the smallest ones for DAVP (5–6 Å). The respective values for both *inverso*-analogues are about 7 Å. In turn, the dihedral angle values, *Ang*_Tyr–Phe_, cluster about roughly perpendicular planes, *Ang*_Tyr–Phe_ = −100°, 20°, 110° and 100° for AVP, DAVP, iAVP and iDAVP, respectively, where the given angles are the average values in the cluster. As seen, the AVP and iAVP have a similar *Ang*_Tyr–Phe_ values, but with opposite signs. This relationship does not hold for DAVP and its *inverso* analogue. The differences in the *Ang*_Tyr–Phe_ values arise mainly from the change of amino acid configuration from l to d, which evidently alters the side chain orientation. Even the change of configuration of one amino acid, Arg^8^, has a significant impact on the increase of the *Ang*_Tyr-Phe_ dihedral angle value (see DAVP). The different arrangements of the Tyr^2^ and Phe^3^ side chains, crucial for activities of the vasopressin-like peptides, are thought to be responsible for diverse activities of the peptides.

In summary, all the peptides show a strong tendency to create typical of vasopressin-like peptides *β*-turns—in positions 3, 4 and/or 4, 5. Therefore, their different activities can be interpreted in terms of different arrangements of the crucial for vasopressin-like peptides activities side chains (of Tyr^2^ and Phe^3^).

### Radial distribution functions and hydration numbers for AVP and its analogues in the mixed anionic–zwitterionic micelles

The radial distribution functions (RDFs) were computed using all the amino acid residues in each peptide (Figs. 9S, 10S in supplementary material). Also, the hydration numbers were calculated to investigate the interactions with aqueous environment (Table. 2S in supplementary material). In AVP and DAVP aromatic Phe^3^ is deeply immersed in the hydrophobic core of the micelle, in contrast to their *inverso* analogues, as additionally confirmed by low values of hydration number calculated for its side chain. Therefore, the reason of the low NH temperature coefficient of this residue might be the shielding by the micelle from exchange with the solvent, rather than an intramolecular hydrogen bond. The Tyr^2^ side chain of AVP, iAVP and DAVP is not deeply immersed in the micelle’s core, while in iDAVP, this residue is exposed to the aqueous environment.

The positively charged center on the Arg side chain of iAVP, DAVP and iDAVP shows the tendency to associate with head groups of the micelle, in contrast to AVP in which the Arg^8^ side chain is markedly exposed to the aqueous phase. On the other hand, the backbone carbonyl oxygen of Arg^8^ of each peptide is strongly hydrated. The side chains of residues 1 and 6 of each peptide are evidently not embedded into the micelle core, resulting in disulphide bridges exposed to the aqueous phase.

## Conclusions

As biological activity of vasopressin-like peptides results from several features, such as conformation, appropriate orientation of the side chains and the presence of a positively charged residue at position 8 (Walse et al. 1998; Langs et al. 1986; Ślusarz et al. 2006b; Postina et al. 1996; Hruby et al. 1979), we analyzed all of them. Moreover, our results suggest that the presence of liposomes induces conformational changes in vasopressin-like peptides. They partially restrict conformational freedom of the peptides and probably induce conformations resembling those of biologically relevant ones.

The results have shown that AVP and DAVP tend to adopt *β*-turns in the 2–5 and 3–6 fragments, which is typical of vasopressin-like peptides (Liwo et al. 1996). The common conformational feature of the *inverso*-analogues, iAVP and iDAVP, is a high propensity to create the 4,5 and 7,8 *β*-turns. Moreover, our study has shown that the Arg^8^ side chain in AVP weakly interacts with the micelle head groups and is strongly hydrated, suggesting the guanidinium group of Arg^8^ to be fairly well exposed to the water environment. These suggestions are well founded when regarding the fact that the Arg^8^ guanidinium group interacts with the extracellular (EL2) loop of V2 receptor and accordingly is exposed to the entrance of the binding pocket (Ślusarz et al. 2006a), in agreement with its protrusion to the hydrophilic environment. In the case of remaining analogues, the Arg^8^ side chain interacts with micelle’s surface. It is worth mentioning that the inversion of Arg^8^ from l to d apparently alters the side chain orientations of Tyr^2^ and Phe^3^, these being vital for interactions whit the receptors.

In the presence of the liposomes, the conformational changes of the peptides are observed. The smallest conformational changes are noticed for DPPC liposomes and the largest for DPPG liposomes, this suggesting that electrostatic interactions are crucial for the peptide–membrane interactions. The shape of the CD spectra of the peptides in the presence of the micelles also differentiated AVP and DAVP, and their *inverso* analogues. This suggests that all the peptides interact differently with the different micelles. Moreover, the smallest differences were found with the zwitterionic DPC micelle, likewise as in the presence of the zwitterionic DPPC liposomes. The similar CD spectra were obtained for the mixed DPC/SDS micelles (5:1) and the mixed DPPC/DPPG (7:3) liposomes as well.

The temperature affects the shape of the CD spectra through changes in organization of the liposomes, and in consequence also through changes in peptides–liposomes interactions and in conformations of the peptides in lipid solutions. In the zwitterionic DPPC liposomes, below the temperature of the main phase transition [*T*m = 41.4 °C (Garidel et al. 1997)], in the gel phase of phospholipids, most of all the hydrophobic interactions with the peptides can be expected. Above *T*m, in the liquid crystalline phase, the negatively charged phosphatidyl groups are more exposed (Chen and Tripp 2012). Consequently, the electrostatic interactions between the positively charged C-terminal part of the peptides and negatively charged phosphatidyl groups are likely to occur. In the DPPG and mixed DPPC/DPPG liposomes, the hydrophobic interactions also occur, but the presence of anionic DPPG adds up negative charges and additional strong electrostatic interactions can be seen even below *T*m [*T*m = 42, 41.5 and 40 °C for DPPC/DPPG (9:1), DPPC/DPPG (7:3) and DPPG, respectively (Garidel et al. 1997)]. The electrostatic interactions may affect the conformation of the C-terminal part of the peptide. Moreover, inspection of the CD spectra suggests different mutual arrangement of the aromatic side chains of Tyr^2^ and Phe^3^ in DAVP in liposomes than in AVP, which can explain a potent antidiuretic activity and enhanced selectivity of this peptide.

We suggest that the differences in the structures and interactions of the peptides with the micelles and the liposomes explain the lack of activities of the *inverso*-analogues. Our results shed a new light on potential roles played by cell membrane for adopting active conformations of vasopressin-like peptides.

## Electronic supplementary material

Supplementary material 1 (DOC 5540 kb)

## Abbreviations

AVP: Arginine-vasopressin
CSDS: Cambridge Structural Database System
DAVP: [d-Arg^8^]-vasopressin
DPC: Dodecylphosphocholine
DPPC: 1,2-Dipalmitoyl-*sn*-glycero-3-phosphatidylcholine
DPPG: 1,2-Dipalmitoyl-*sn*-glycero-3-phosphatidylglycerol
DSS: 2,2-Dimethyl-2-silapentanesulfonic acid
GPCR: G-protein coupled receptor
Iavp: *Inverso*-vasopressin
iDAVP: *Inverso*-[d-Arg^8^]-vasopressin
MD: Molecular dynamics
NPH: Neurohypophyseal hormone
SDS: Sodium dodecyl sulphate
TAV-MD: Time-averaged-MD
Tm: Temperature of the main phase transition
