# Supplementary material for: Arginine-, d-arginine-vasopressin, and their inverso analogues in micellar and liposomic models of cell membrane: CD, NMR, and molecular dynamics studies
Source: Eur Biophys J. 2015 Aug 20;44(8):727–43. doi: 10.1007/s00249-015-1071-4 (PMC4628624; doi:10.1007/s00249-015-1071-4)
Supplement: Supplementary file 1 — Supplementary material 1 (DOC 5540 kb) [file 249_2015_1071_MOESM1_ESM.doc]

**~~NMR structure and interactions with model lipid bilayers of arginine- and D-arginine-vasopressin, and their~~ *~~inverso~~* ~~analogues~~**

**Arginine-, D-arginine-vasopressin, and their *inverso* analogues in micellar and liposomic models of cell membrane: CD, NMR and molecular dynamics studies**

Emilia A. Lubecka^1^*, Emilia Sikorska^1^, Dariusz Sobolewski^1^, Adam Prahl^1^, Jiřina Slaninová^2^ and Jerzy Ciarkowski^1^

1. Faculty of Chemistry, University of Gdańsk, 80-308 Gdańsk, Poland

2. Institute of Organic Chemistry and Biochemistry, Academy of Sciences of the Czech Republic, 166 10 Prague, Czech Republic

*Corresponding author: Emilia Lubecka, Faculty of Chemistry, University of Gdańsk, Wita Stwosza 63, 80-308 Gdańsk, Poland, T:+48 58 523 5351, email: *emilial@chem.univ.gda.pl*

**Table 1S.** Pharmacological properties of the peptides studied.

| **Analogue** | **Activities** | | |
| --- | --- | --- | --- |
|  | **Oxytocic uterus *in vitro***  **no Mg^2+^ IU/mg (pA_2_)** | **Pressor IU/mg (pA_2_)** | **Antidiuretic IU/mg**  **60 min** |
| **AVP ^a^** | 17 | 412 | 465 |
| ***inverso*-AVP** | 0 | 0 | 0 |
| **[D-Arg^8^]-VP ^a^** | 0.4 | 4.1 | 114-257 ^b^ |
| ***inverso*-[D-Arg^8^]-VP** | 0 | 0 | 0 |

a – Values taken from ref. (Lebl et al. 1987).

b – Values taken from ref. (Slaninová 1987).

Lebl M, Jost K, Brtnik F (1987) Tables of Analogs. In: Handbook of Neurohypophyseal Hormone Analogs. CRC Press Inc., Boca Raton, Florida, pp 127–267.

Slaninová J. (1987) Fundamental Biological Evaluation. In: Handbook of Neurohypophyseal Hormone Analogs, CRC Press Inc., Boca Raton, Florida, pp 83–107.

**Table 2S.** Hydration numbers for each residue in each analyzed peptide in the mixed anionic-zwitterionic micelle. The hydration numbers were averaged over the finally ensembles for each peptide.

| **Residue** | **Carbonyl oxygen** | | | | **Side chains** | | | |
| --- | --- | --- | --- | --- | --- | --- | --- | --- |
|  | **AVP** | **iAVP** | **DAVP** | **iDAVP** | **AVP** | **iAVP** | **DAVP** | **iDAVP** |
| **L-/ D-Cys^1^** | 0.63 | 0.62 | 0.87 | 0.70 | 0.47 | 0.36 | 0.55 | 0.22 |
| **L-/ D-Tyr^2^** | 0.08 | 0.45 | 0.12 | 0.63 | 0.26 | 0.20 | 0.18 | 0.52 |
| **L-/ D-Phe^3^** | 0.00 | 0.30 | 0.21 | 0.67 | 0.03 | 0.33 | 0.03 | 0.39 |
| **L-/ D-Gln^4^** | 0.69 | 0.47 | 0.51 | 0.72 | 0.22 | 0.29 | 0.14 | 0.55 |
| **L-/ D-Asn^5^** | 0.38 | 0.30 | 0.66 | 0.34 | 0.62 | 0.37 | 0.55 | 0.65 |
| **L-/ D-Cys^6^** | 0.40 | 0.20 | 0.83 | 0.50 | 0.16 | 0.13 | 0.31 | 0.29 |
| **L-/ D-Pro^7^** | 0.70 | 0.40 | 0.53 | 0.31 | 0.11 | 0.20 | 0.22 | 0.04 |
| **L-/ D-Arg^8^** | 0.67 | 0.72 | 0.81 | 0.56 | 0.61 | 0.30 | 0.39 | 0.29 |
| **Gly^9^** | 0.94 | 0.99 | 0.87 | 0.88 | - | - | - | - |


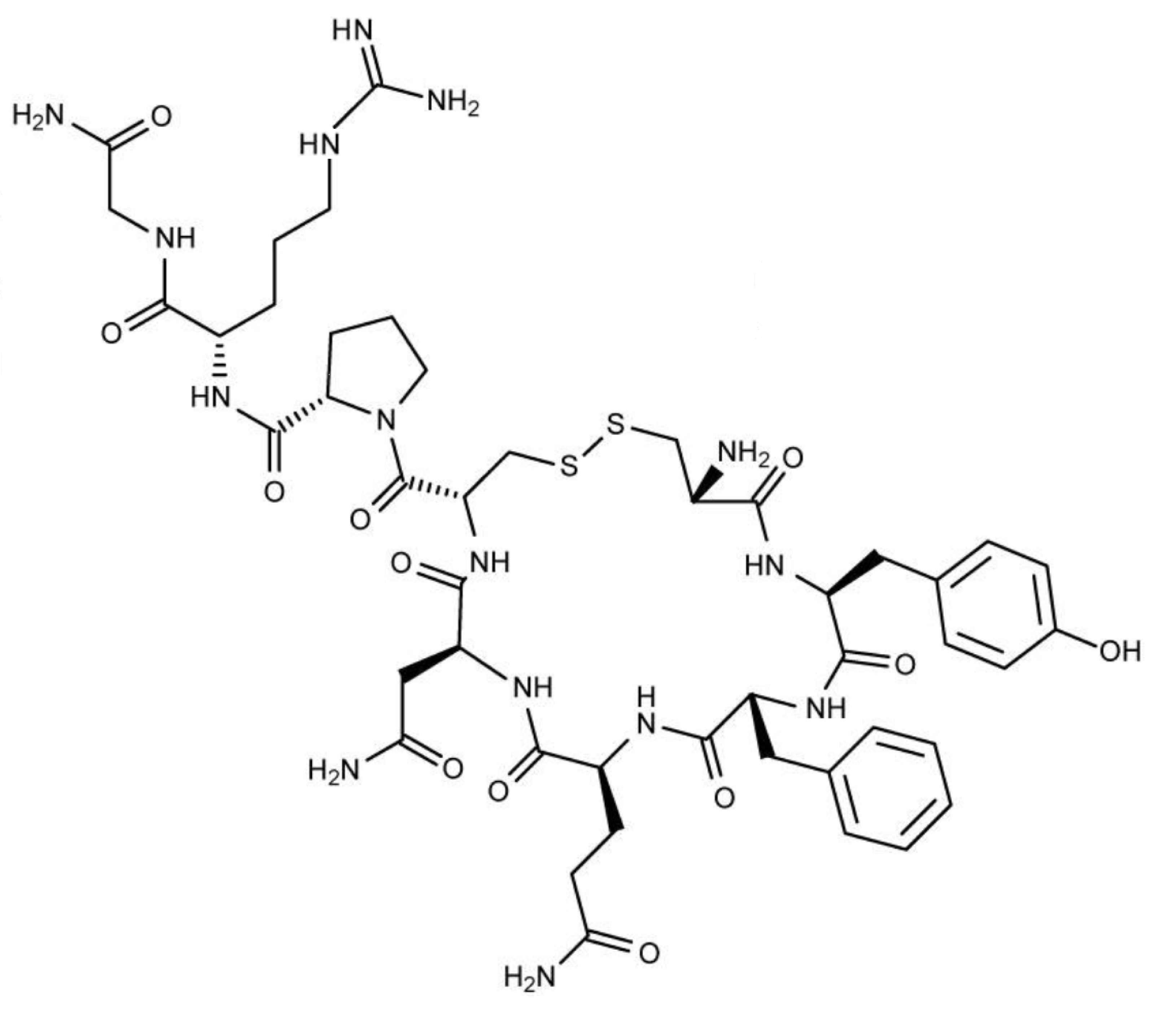


**Figure 1S.** Structure of arginine-vasopressin (AVP).

**Figure 2S.** The initial anionic-zwitterionic micelle / water / AVP system configuration, and the syste
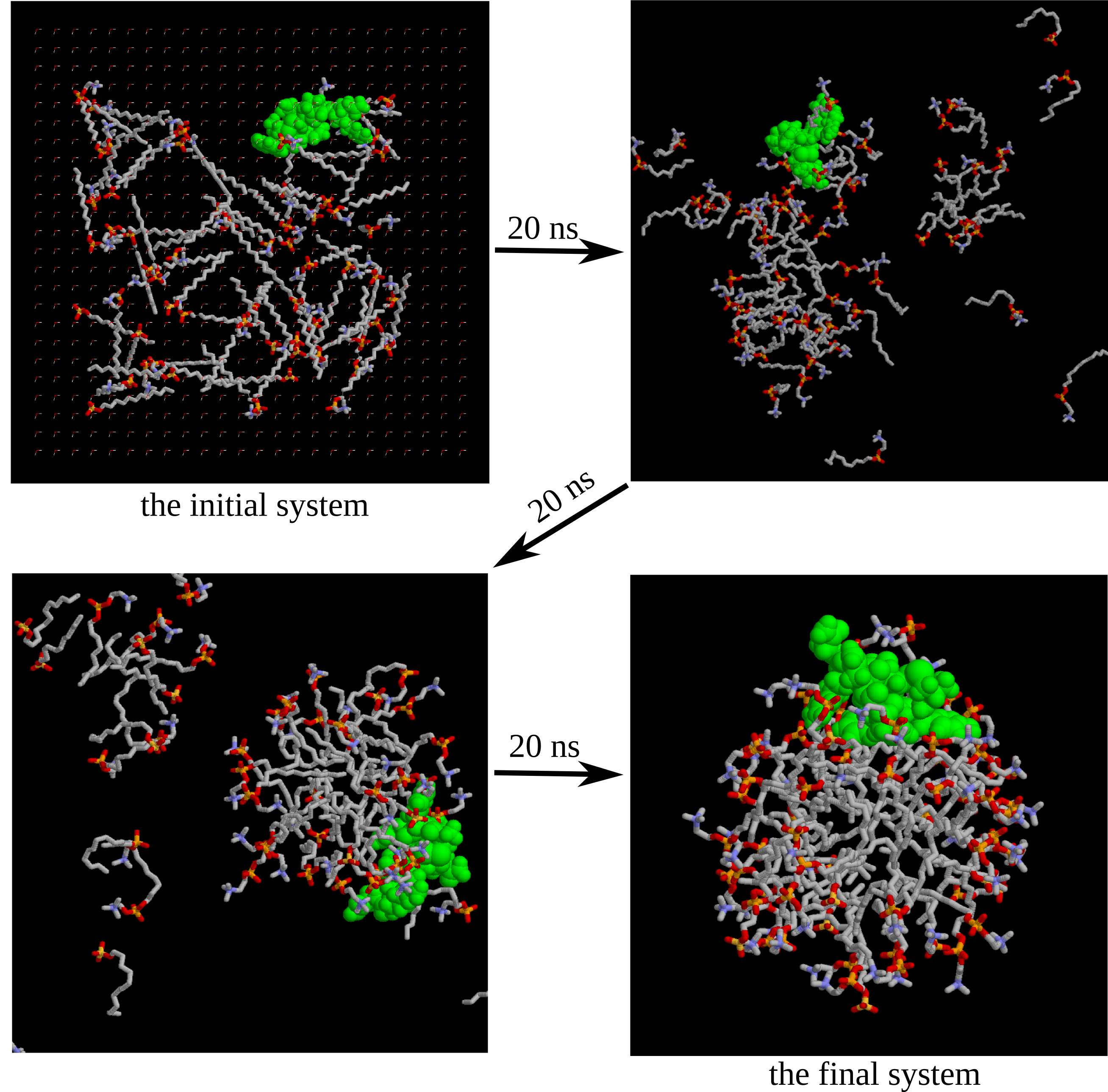
m after: 20 ns, 40 ns and 60 ns of molecular dynamics simulations. The water molecules in the systems after 20 ns, and more, are not shown for clarity. The pictures were made using RASMOL (Sayle and Milner-White 1995).

Sayle R, Milner-White JE (1995) RASMOL: biomolecular graphics for all. TIBS 20:374–376. doi: 10.1016/S0968-0004(00)89080-5.


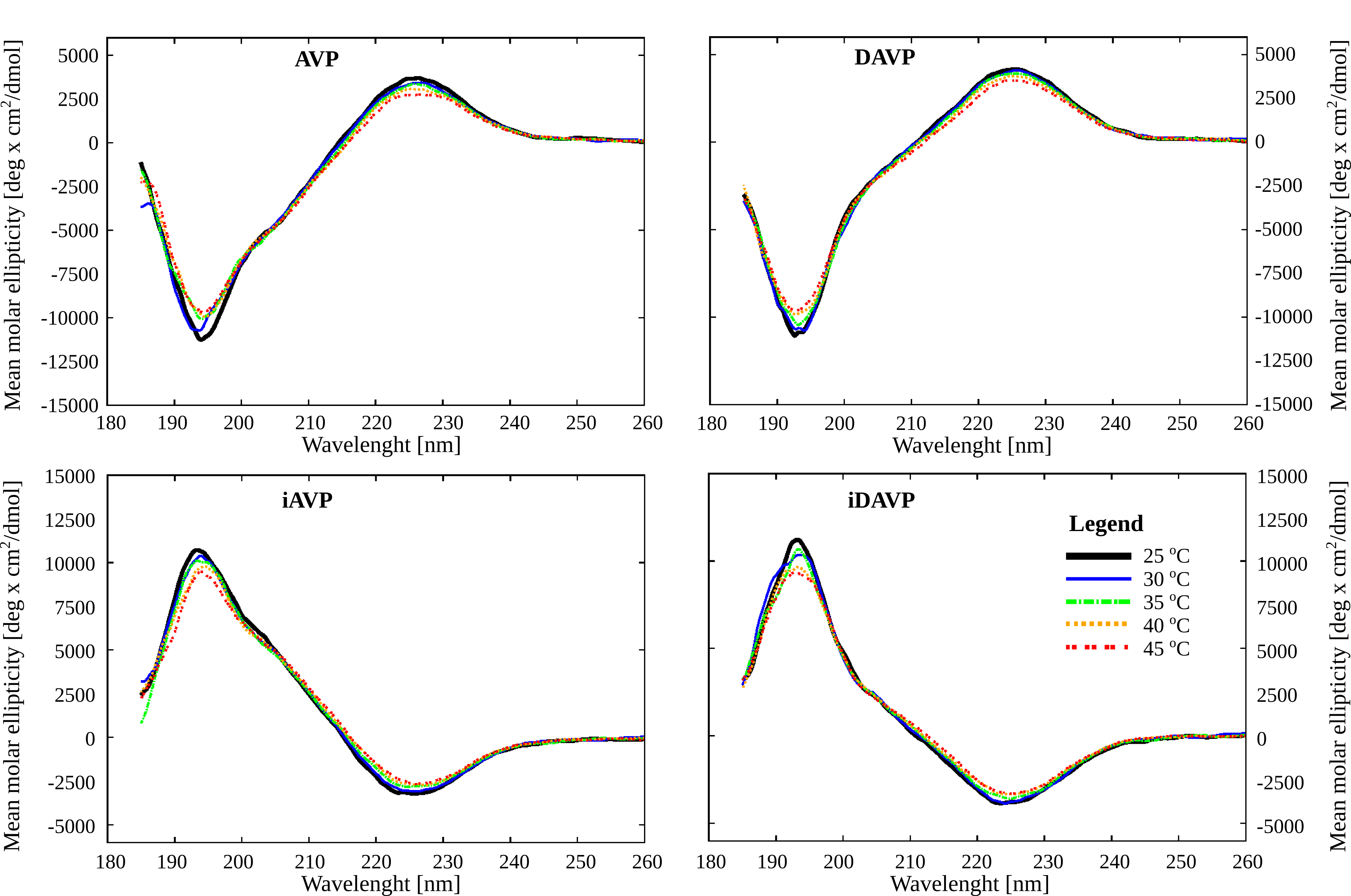
**Figure 3S.** The temperature dependence of the far-UV CD spectra of the peptides in the lipid-free solution (water), where AVP: arginine-vasopressin, iAVP: *inverso*-AVP, DAVP: [D-Arg^8^]-AVP and iDAVP: *invers*o-DAVP.

**Figure 4S.**
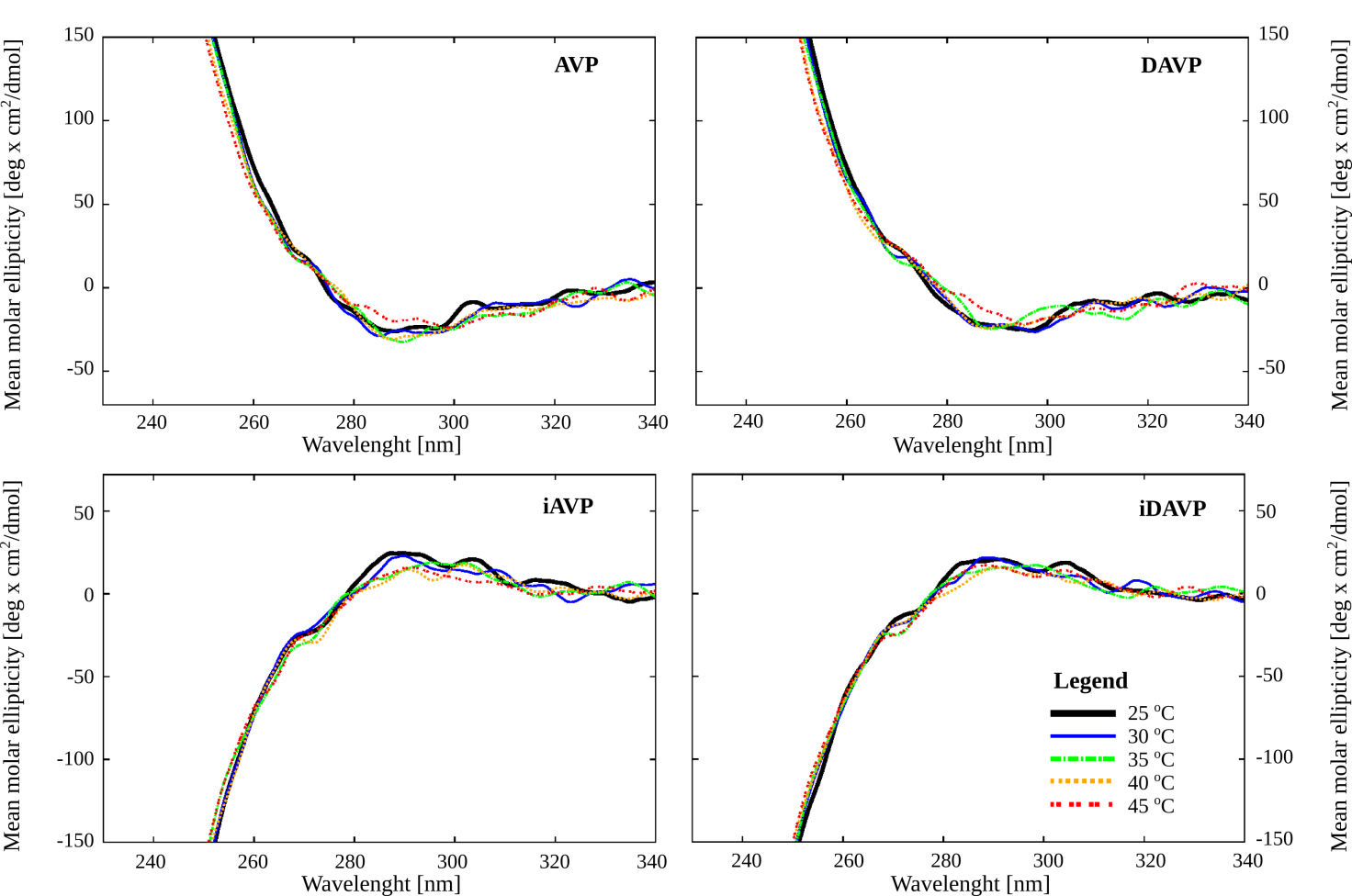
 The temperature dependence of the near-UV CD spectra of the peptides in the lipid-free solution (water), where AVP: arginine-vasopressin, iAVP: *inverso*-AVP, DAVP: [D-Arg^8^]-AVP and iDAVP: *invers*o-DAVP.


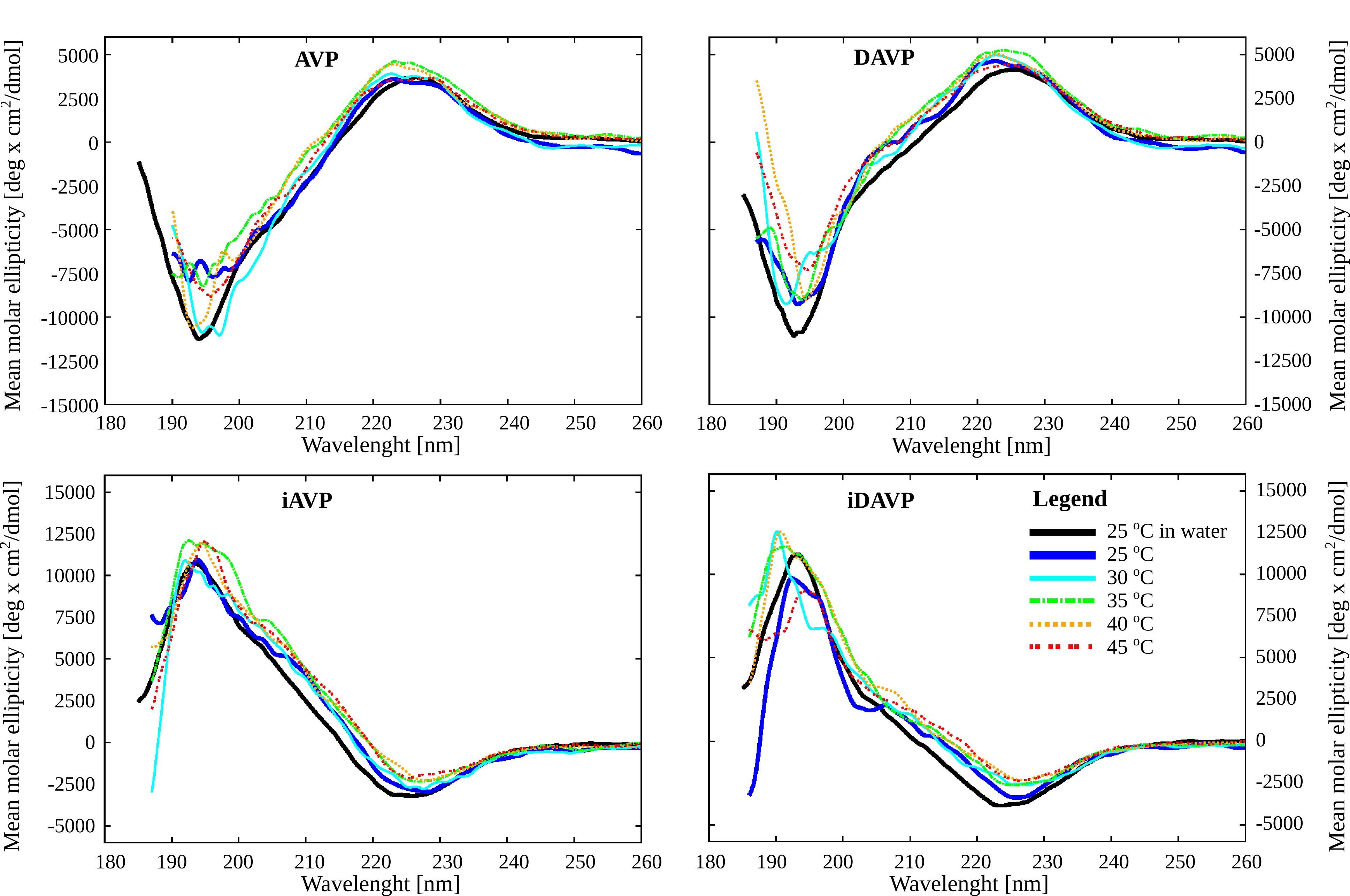
**Figure 5S.** The temperature dependence of the far-UV CD spectra of the peptides in the presence of DPPC liposomes, where AVP: arginine-vasopressin, iAVP: *inverso*-AVP, DAVP: [D-Arg^8^]-AVP and iDAVP: *inverso*-DAVP.


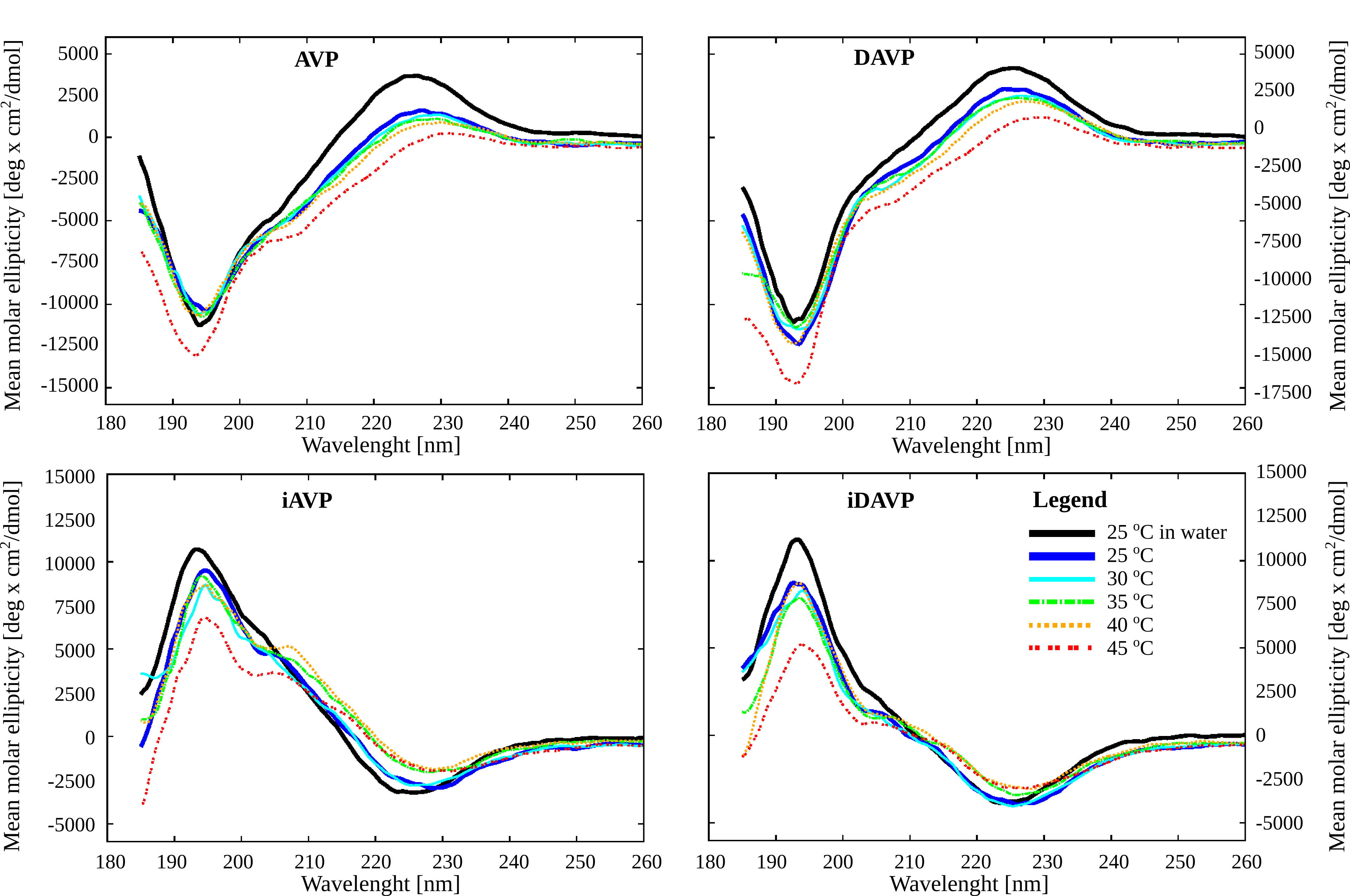
**Figure 6S.** The temperature dependence of the far-UV CD spectra of the peptides in the presence of DPPC/DPPG (9:1, mol:mol) liposomes, where AVP: arginine-vasopressin, iAVP: *inverso*-AVP, DAVP: [D-Arg^8^]-AVP and iDAVP: *inverso*-DAVP.


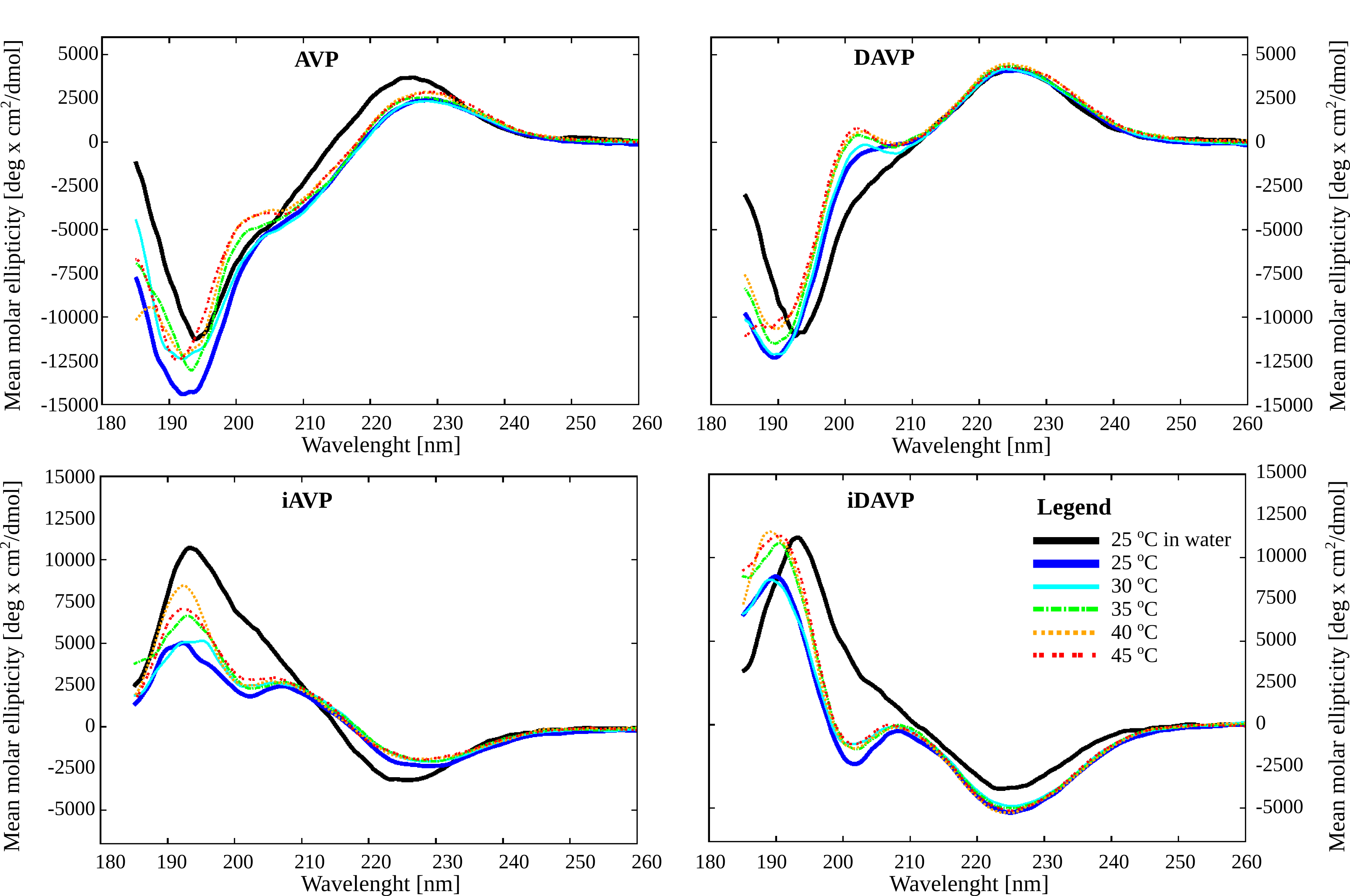
**Figure 7S.** The temperature dependence of the far-UV CD spectra of the peptides in the presence of DPPG liposomes, where AVP: arginine-vasopressin, iAVP: *inverso*-AVP, DAVP: [D-Arg^8^]-AVP and iDAVP: *inverso*-DAVP.


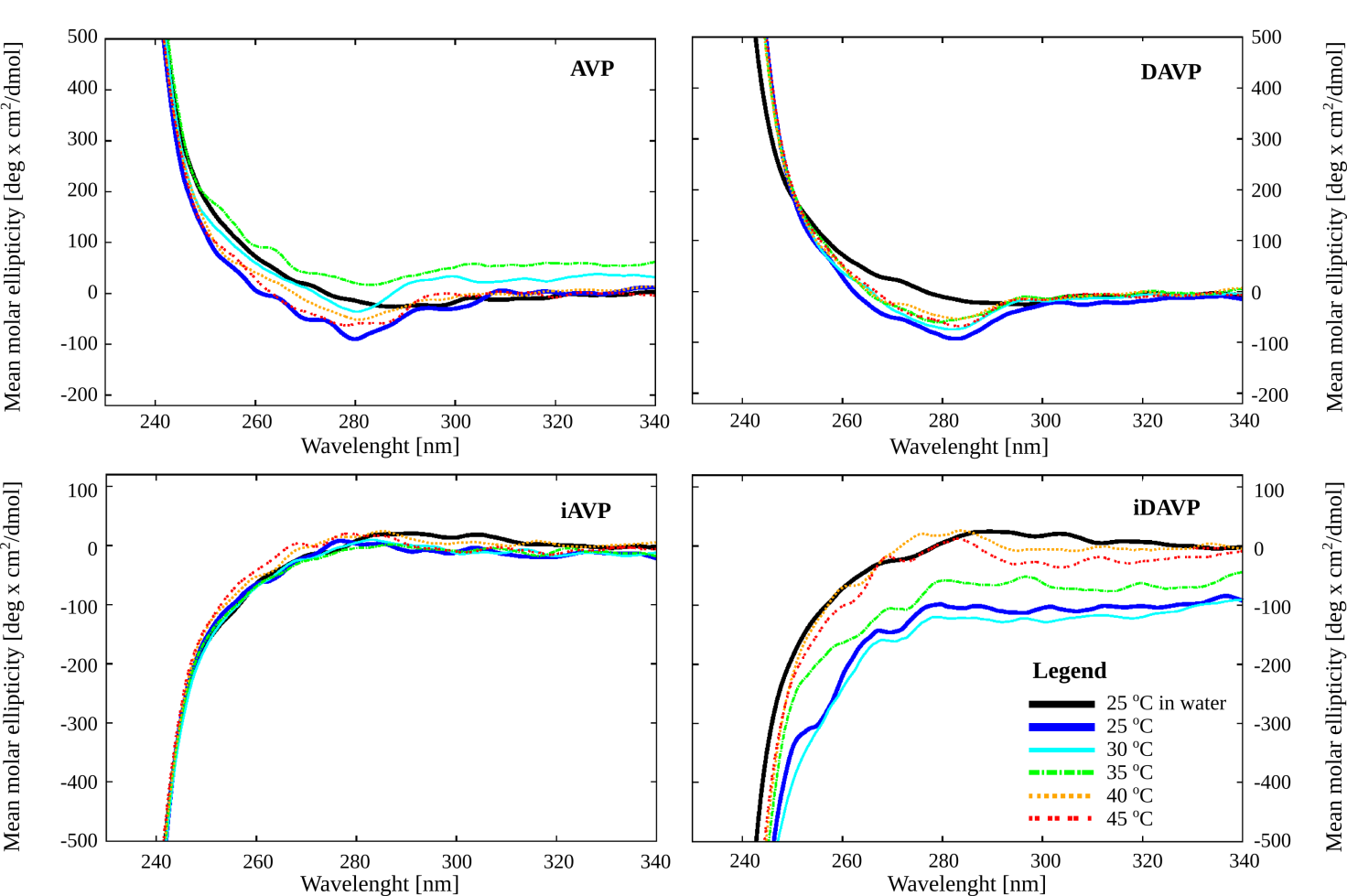
**Figure 8S.** The temperature dependence of the near-UV CD spectra of the peptides in the presence of DPPC/DPPG (7:3, mol:mol) liposomes, where AVP: arginine-vasopressin, iAVP: *inverso*-AVP, DAVP: [D-Arg^8^]-AVP and iDAVP: *inverso*-DAVP.

**~~Figure 5S.~~** ~~The fingerprint regions of the TOCSY spectra of AVP (black) and DAVP (gray) showing the correlation between the amide protons and the side-chain protons.~~

**Figure 9S.**
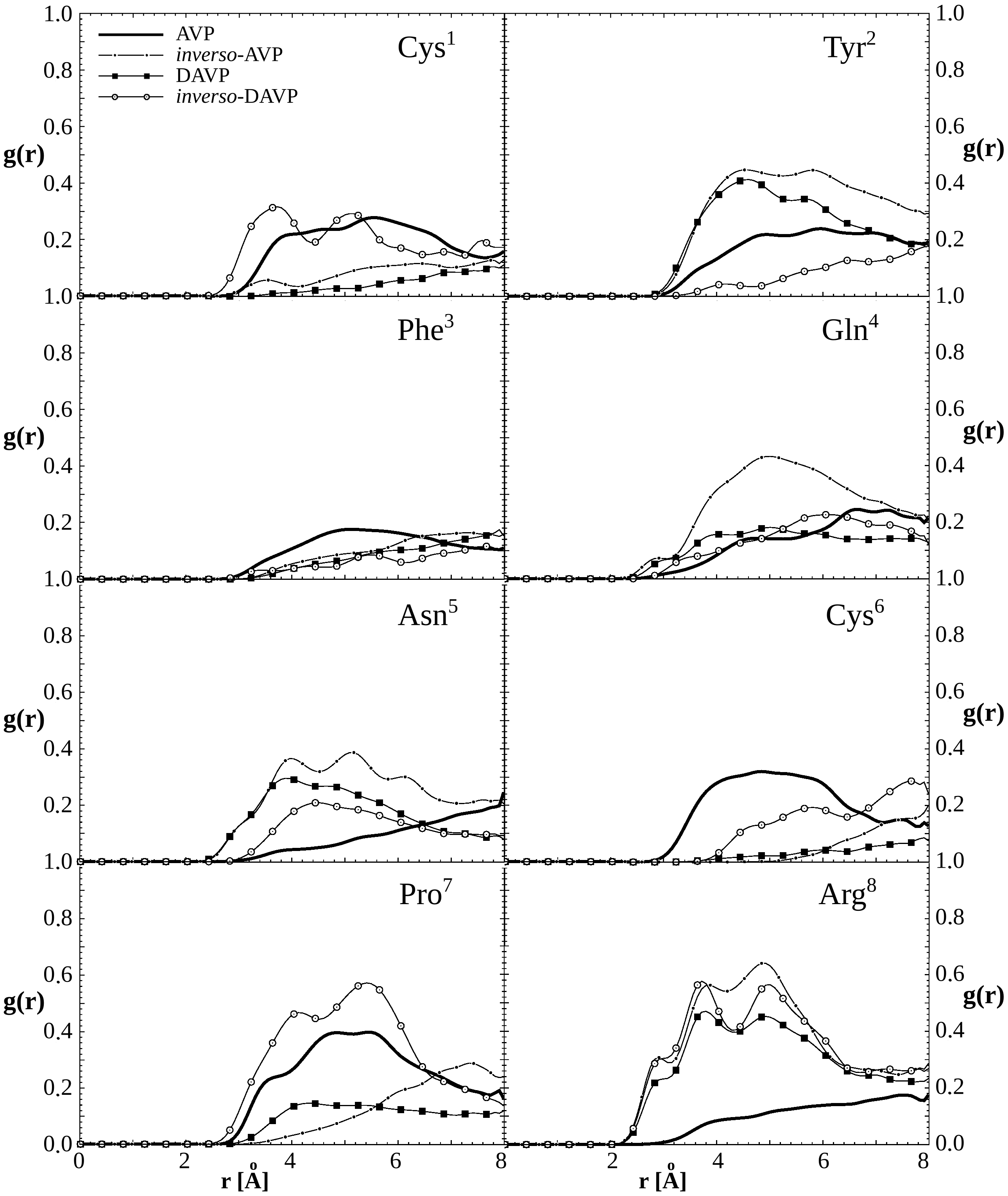
 Radial distribution functions g(r) between the micelle head groups and the side chain (heavy atoms) of each residue of native AVP, *inverso*-AVP, DAVP and *inver**so*-DAVP. The r parameter defines the distance in Å.

**Figure 10S.**
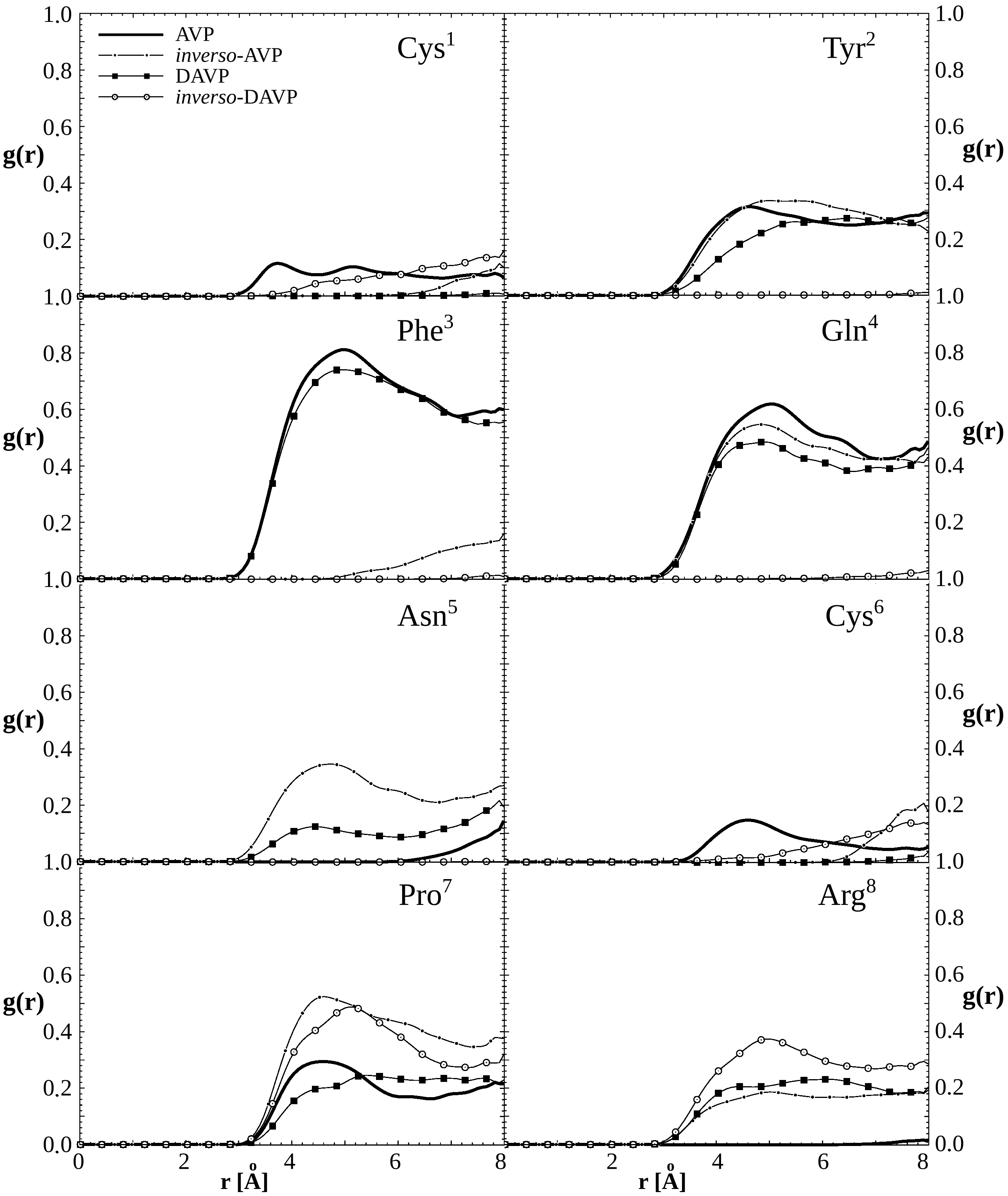
 Radial distribution functions g(r) between the hydrophobic micelle core and the side chain (heavy atoms) of each residue of native AVP, *inverso*-AVP, DAVP and *inverso*-DAVP. The r parameter defines the distance in Å.
